# Does language prestige hypothesis hold true across typologically distant languages? A corpus-based study comparing shining-through effect in English-Chinese bidirectional translations

**DOI:** 10.1371/journal.pone.0353687

**Published:** 2026-07-22

**Authors:** Jia Li, Yunhao Pan

**Affiliations:** 1 School of Foreign Languages, Wuhan University of Technology, Wuhan, China; 2 School of Foreign Languages, Hubei University, Wuhan, China; Kazan University, RUSSIAN FEDERATION

## Abstract

While the influence of language prestige on the source language shining through has been well-documented in studies involving typologically similar languages, its impact on typologically distant language pairs remains underexplored. This article examines the interplay between language prestige and typological differences in shaping the shining-through effect, extending the analysis to Chinese-English bidirectional translations. Based on a bidirectional parallel balanced corpus, the study compares the shining-through effect in both Chinese-to-English and English-to-Chinese translations through the application of principal component analysis and flexible discriminant analysis. The findings revealed that the shining-through effect was present in both translation directions but was more pronounced in English-to-Chinese translations. Additionally, the effect varied across registers, with imaginative texts showing a stronger tendency than the informative texts. These results provide further evidence supporting the hypotheses of language prestige, typological distance and risk aversion. A comparison with findings from studies involving typologically closer language pairs further suggests that typological distance may help explain variation in the influence of language prestige on source language shining through. Specifically, the prestige effect appears to be weaker when the source and target languages are typologically more distant, although this interpretation requires direct empirical testing in future research.

## 1. Introduction

The distinctive linguistic features that set translated texts apart from originally authored works have been variously conceptualised as the “third code” [1] or “translationese” [2]. It was Baker [3, 243], however, who introduced the more systematic notion of translation universals to denote those linguistic properties considered intrinsic to translated language. In her formulation, translation universals are defined as “features which typically occur in translated text rather than original utterances and which are not the result of interference from specific linguistic systems”. This theoretical framework has since guided a substantial body of empirical research, with scholars examining linguistic variation between translated and non-translated corpora. To date, a number of universal candidates have been proposed and subjected to scrutiny, including explicitation, simplification, normalisation, shining-through, convergence and unique items (e.g., [4–13]).

Of particular theoretical interest among these universals are normalisation and shining-through, which occupy opposite poles of a continuum measuring the degree to which a translated text gravitates toward the target language or retains traces of the source language [13]. Normalisation denotes the tendency of translations to “conform to patterns and practices which are typical of the target language, even to the point of exaggerating them” [4]. Conversely, source language shining through constitutes a competing force that draws the translated text toward the structural and stylistic conventions of the source language [13]. Empirical evidence for the shining-through effect has been documented across a range of language pairs, including French-English [6,7], English-Russia [14], German-English [8,11] and Finnish-English/Russian [15].

A comprehensive review of the existing literature indicates that the shining-through effect has been well-documented in the context of English-to-Chinese translation. Xiao [16] demonstrated that passive constructions occurred with greater frequency in translated Chinese texts across different genres, a finding interpreted as indicative of shining-through. Building on this line of inquiry, Dai and Xiao [17] examined passive voice usage in translated Chinese through the analysis of two comparable and two parallel corpora, concluding that source language shining through tended to manifest more prominently in non-literary registers. Zhang et al. [18] investigated the deployment of personal pronouns in English-to-Chinese translations of children’s literature, observing a heightened rate of explicit pronominal usage in the translated texts, which the authors attributed to the influence of shining-through. Pang and Wang [19] adopted a diachronic perspective, drawing on a corpus spanning the twentieth century through the 1990s to trace the evolving impact of source language shining through on the explicitness of adversative conjunctions in English-to-Chinese translations. Employing a news corpus, Hu and Kübler [9] conducted a systematic analysis of the linguistic properties of translated Chinese produced from multiple source languages, revealing that translations from Altaic languages bore discernible traces of source language shining through, as evidenced by distinctive patterns in nominal and pronominal usage.

Notwithstanding the valuable contributions of these studies, a number of significant questions remain insufficiently addressed. Prior scholarship has posited that the shining-through effect is amplified when translation proceeds from a more dominant to a less dominant language, and when the languages involved exhibit substantial typological divergence (see Sections 2.1 and 2.2 for an extended discussion). The studies reviewed above have consistently identified a pronounced shining-through effect in English-to-Chinese translations, a finding attributable to the considerable typological distance between the two languages and the directionality of translation from a higher- to a lower-status language. The dynamics of translation in the reverse direction, from Chinese into English, a language that occupies the position of a global lingua franca, present a markedly different set of conditions. In this case, the translational movement proceeds from a lower- to a higher-status language, which raises the question of whether the typologically induced shining-through effect is attenuated by the institutional prestige and global dominance of the target language.

A further issue that remains less addressed in previous studies is their reliance on univariate analytical methods, register-controlled designs and exclusively monolingual comparable corpora. Whilst univariate approaches offer a useful means of examining individual linguistic features in translated versus non-translated texts, they lack the explanatory capacity required to account for the systemic properties of language varieties [8,20]. The failure to treat register as a variable shaping the linguistic character of translated texts has been highlighted by Delaere and De Sutter [21] and Delaere et al. [22], who contend that the universal tendencies observed in translated language are substantially register-dependent. The exclusive reliance on monolingual comparable corpora, which, by definition, exclude the corresponding source texts, further constrains the analytical scope, as some of the observed differences between translated and non-translated texts may be attributable to properties of the source texts rather than to the translation process itself [23]. As Evert and Neumann [8] have emphasised, a systematic comparison between translated texts and their source counterparts is indispensable for reliably isolating translation-specific features. This position is corroborated by Teich [13], who argues that the detection of normalisation and shining-through effects necessitates the inclusion of both source and target language data in order to disentangle these phenomena from other translationese-related influences. Compounding these methodological difficulties is the considerable typological distance between Chinese and English, which renders the identification of cross-linguistically comparable features a particularly complex undertaking.

In response to these methodological concerns, the present study identifies 34 linguistic indicators that are both theoretically applicable and computationally extractable across Chinese and English, and adopts the semi-supervised multivariate framework developed by Diwersy et al. [24]. This framework integrates dimensionality reduction and visualisation techniques, thereby enabling a nuanced and systematic investigation of cross-linguistic variation and contrast. The study pursues two overarching objectives: to explore and corroborate the shining-through effect across two translational directions (Chinese-to-English and English-to-Chinese) and to examine the extent to which source language shining through manifests across different registers within each direction. These objectives are operationalised through three specific research questions: (I) Do translated texts in English and Chinese exhibit statistically distinguishable patterns relative to non-translated texts in the respective languages? (II) Do English-to-Chinese translations display a stronger orientation toward the source language than translations produced in the reverse direction? (III) Are these source language tendencies consistently observable across a range of registers? In addressing these questions, the study constructs a classification model capable of detecting the shining-through effect in Chinese–English translation pairs through a multivariate analytical lens. At a broader level, the study aims to provide empirical evidence that the linguistic properties of translated texts reflect the complex interplay of typological, sociolinguistic and register-related factors.

## 2. Related work

### 2.1. Language prestige hypothesis

The first theory closely related to the present study is the language prestige hypothesis. In his formulation of the two laws (the “law of interference” and the “law of growing standardisation”), Toury [25, 275–276] describes how textual features from the source language are transferred or adapted to the target language. The law of interference, which is conceptually akin to the shining-through effect, operates in contrast to the law of growing standardisation, which involves modifying the translated text to better align with the norms of the target language or culture. In this context, Toury [25, 278] also introduced the language prestige hypothesis: when the text is translated from a less dominant language to a more prestigious one, the translation tends to be standardised, with less interference from the source text; however, when the text is translated from a dominant language to a minor one, the translation is more likely to show interference.

Evert and Neumann [8] provided quantitative evidence for this hypothesis in the language pairs of German and English through their application of a machine learning approach, following the methodology outlined by Diwersy et al. [24]. Their study found a more pronounced shining-through effect in translations from English to German than the reverse. They examined the dimension in which the linguistic distance between language varieties was maximised through PCA and linear discriminant analysis (LDA), confirming the hypothesis by observing the distribution of language varieties in this dimension. Specifically, varieties that were linguistically more similar tended to exhibit greater overlap in their density distributions, whereas more distinct varieties showed clearer separation. Evert and Neumann [8] also introduced a distinction between two types of shining-through effects: “genuine” source language shining through and source text interference. The former refers to the tendency of translators to introduce features typical of the source language into the target language, while the latter involves the transfer of text-specific characteristics such as authorial style, tone or thematic features from the source text to the target. In this case, the effect is considered interference from the source text. A similar conclusion was reached by Van Oost et al. [26], who identified significant differences in the placement of prepositional phrases in German-Dutch and Dutch-German translations. They suggested that German, being the more prestigious language, exerted a stronger influence in the Dutch translations, which featured more middle-positioned prepositional phrases, a characteristic of German usage, compared to non-translated Dutch.

Nevertheless, the findings in these studies may be attributed to the greater typological similarity between the source and target languages, which makes the prestige effect more likely to be observed. Therefore, the central question for the present study is whether the language prestige hypothesis remains valid when examining language pairs that are typologically distant from one another. Addressing this question requires consideration of another important explanation for the shining-through effect, namely the typological distance itself.

### 2.2. Typological distance hypothesis

While the language prestige hypothesis attributes variation in shining-through to asymmetries in the sociolinguistic status of languages, another line of research emphasises the role of structural differences between languages. This perspective is commonly referred to as the typological distance hypothesis, which can be summarised as follows: the shining-through effect is likely to become more pronounced when there is a greater typological divergence between the source and target languages. It is important to note that although it was Teich [13] who introduced the concept of source language shining through and established its connection to typological differences, this hypothesis was formulated by a series of following studies.

The role of typological difference in the formation of source language shining through was demonstrated by Cappelle and Loock [7], who observed that translations from Germanic languages to English tended to use phrasal verbs more frequently than translations from Romance languages. By comparing the frequency of phrasal verbs such as *up*, *out* and *down* in translations from different source languages, they argued that Romance languages, due to their less close genetic relationship with English, offered fewer direct equivalents, leading to stronger shining-through effects. Similarly, Kunilovskaya et al. [27] explored the influence of typological differences in Russian translated literature, finding that translationese effects were more prominent in translations from typologically distant languages. In line with this, Nikolaev et al. [28] studied the morphosyntactic predictability of translations from various source languages, and found that translations from structurally similar languages often displayed mixed syntactic patterns, whereas those from highly divergent languages exhibited more unpredictable syntactic features. Rabinovich et al. [29] further validated the shining-through effect, asserting that interference from the source language was a key feature of translated texts. Their analysis of 14 languages from three different language families successfully reconstructed phylogenetic language trees using monolingual texts. In a similar vein, Dutta Chowdhury et al. [30] included non-Indo-European languages in their study and found evidence of source language shining through, supporting the hypothesis of typological distance through the Gromov-Hausdorff distance metric.

Nevertheless, in certain cases, the impact of typological difference may compete or conflict with the influence of language prestige. For instance, in this study, translating from Chinese to English presents a situation where the translation direction involves a shift from a less dominant to a more prestigious language. The language prestige hypothesis, if applicable, would predict a reduction in the shining-through effect or even a total elimination, but the typological distance between the two languages may conversely result in a very noticeable shining-through effect. Hence, it remains uncertain whether the significant typological distance between Chinese and English would diminish or inhibit the influence of the prestige effect. Moreover, even when language prestige and typological distance are taken into account, the manifestation of shining-through may vary considerably across communicative contexts and registers.

### 2.3. Register variation and risk-aversion hypothesis

Beyond language prestige and typological distance, studies have also highlighted the influence of register-specific effects on translation patterns. For example, Lapshinova-Koltunski [11] explored the role of register by using a support vector machine (SVM) to assess the prevalence of either normalisation or shining-through effects. Lapshinova-Koltunski [11] evaluated this by analysing the classification model’s accuracy: if two language varieties were clearly distinct, the algorithm could easily differentiate them, leading to a higher accuracy rate; conversely, more similar varieties would result in greater errors. Although her main goal was to investigate the impact of translator experience on language variation, her findings indicated that the tendencies toward normalisation or shining-through were highly dependent on the register. Specifically, normalisation was found to dominate in literary works, scientific texts and political speeches, whereas shining-through was more apparent in political essays, instructional materials and tourism leaflets. Similarly, Kunilovskaya and Cortpas Pastor [14] confirmed the impact of register on the shining-through effect using PCA and SVM. Their results suggested that general media texts tended to show a stronger inclination towards shining-through, while fictional and journalistic texts displayed more normalisation.

The variability of tendencies towards normalisation or shining-through aligns with Pym’s [31–33] risk-aversion hypothesis, which posits that translators often make decisions by taking into account the potential linguistic, communicative and social risks in the process of translation. This hypothesis was further supported by Delaere and De Sutter [21], who found that translated Dutch tended to use more standard lexico-grammatical variants compared to original Dutch, with translators making language choices based on the target audience and the specific register of the text. Kruger [34] further supported this hypothesis by examining the differences in the use of the complementiser *that* between translated English from Afrikaans and non-translated English. Her findings revealed that even within fictional texts, a register associated with lower communicative risk, translators tended to explicate *that*. This strategy not only enhances communicative clarity by providing additional cues but also aligns with the conventions of formal written language.

In the context of the present study, if this hypothesis proves valid in the language pairs of Chinese and English, it would be anticipated to observe variations in the degree of similarity between translated texts and their respective source languages across different registers.

### 2.4. The present study

Taken together, previous research suggests that source language shining through is shaped by multiple interacting factors. Existing studies have highlighted the potential roles of language prestige, typological distance and register variation. However, several important questions remain unresolved. First, the relative influence of language prestige and typological distance has rarely been examined in language pairs that are both sociolinguistically asymmetric and typologically distant. Second, little is known about whether the interaction between these factors remains stable across different registers. Finally, most previous evidence has been derived from European language pairs, leaving the generalisability of existing explanations to English–Chinese translation largely unexplored. The present study addresses these gaps by examining shining-through effects in bidirectional English–Chinese translations across multiple registers within a unified analytical framework.

To enhance the interpretability and replicability of findings in such a context, it is necessary to clarify the typological and sociolinguistic profiles of the English–Chinese pair and to specify how the shining-through effect is operationally defined in this study. It should be emphasised that both typological distance and language prestige are best understood as continua rather than fixed categorical distinctions.

Typologically, English and Chinese belong to distinct language families [35]. English is a Germanic language within the Indo-European family, closely related to German and Dutch, and heavily influenced by French, a member of the Romance branch [36]. Despite their genealogical differences, the Germanic and Romance languages share a number of structural features, such as the existence of tense systems, definite and indefinite articles, and the grammatical distinction between singular and plural nouns [37]. These features are either absent or considerably less grammaticalised in Chinese, which belongs to the Sino-Tibetan (Sinitic) family [38]. Furthermore, unlike English, which employs an alphabetic writing system, Chinese is a logographic language that uses Chinese characters as written symbols. As a result, the structural distance between English and Chinese is far greater than that between typologically related European languages, encompassing profound differences in morphology, syntax and information structure, and involving minimal overlap in linguistic encoding strategies [35].

From a sociolinguistic perspective, both English and Chinese can be described as global languages, yet they occupy asymmetrical positions within the world linguistic system. In the present study, language prestige is treated as a theoretically motivated sociolinguistic property of the language pair, rather than as an empirically measured construct. This assumption is grounded in the established sociolinguistic literature on the asymmetric institutional positions occupied by English and Chinese in international communication [39–45]. Crystal [39, 422] defines a global language as one that holds a “special role that is recognised in every country”, while Ammon [40, 101–102] further distinguishes between a language’s global status (geographical coverage), global function (use in international communication) and the factors influencing that function (the sociopolitical and economic motivations for its global use). Although Chinese, in all its varieties, has the largest number of native speakers (over one billion), according to Eberhard [41], its native use is largely geographically concentrated in China [42]. English, by contrast, has a smaller native speaker base across the USA, Canada, Britain, Ireland, Australia, New Zealand, South Africa and parts of the Caribbean, but it enjoys broader global diffusion and institutional presence. As McArthur [43, 414] notes, “the Chinese language complex is much larger than that of native English users, but its members are largely ethnically and culturally homogeneous, and its worldwide distribution is limited”. Despite the growing international influence of Chinese, English continues to occupy a central position as the global lingua franca [44,45].

To visualise and compare the shining-through effect across human and machine translations, the present study adopts the analytical framework of Evert and Neumann [8]. This framework aligns with the multivariate, multifactorial and parallel design advocated by recent research and allows the interplay of linguistic and contextual variables to be systematically modelled. Operationally, the shining-through effect is evidenced when translated texts cluster between the source-language and target-language varieties along the major latent dimensions extracted through multivariate analysis. Such an intermediate positioning indicates that the translated texts retain source-language linguistic patterns to a noticeable degree. By contrast, a displacement toward the monolingual target-language cluster would signal normalisation.

## 3. Data and methods

### 3.1. Corpus

The aim of the present study is to investigate the impact of translation direction on the shining-through effect. To this end, the PolyU Parallel Corpus of Chinese-English Contemporary Texts (PCCE) was employed. This corpus comprises original English, original Chinese, and their respective translated English and Chinese texts [46]. It was constructed based on the design of the Brown family, including four distinct registers, fifteen genres and five hundred texts for each sub-corpus [12]. The PCCE consists of two parallel sub-corpora: the Corpus of Chinese into English (COCE) and the Corpus of English into Chinese (COEC). The bilingual parallel structure of this corpus enables a comprehensive examination of the relationship between source texts and their translated texts in both Chinese-to-English and English-to-Chinese translations.

In the analysis of Ruette et al. [47], the four registers are further categorised into two functional categories: informative and imaginative. The informative category includes genres A-J, covering journalistic, general and academic texts, while the imaginative category sample fictional texts from K to R. This classification may allow for a more simplified approach to understanding how linguistic choices interplay with socio-cultural norms. Table 1 provides a detailed summary of the corpus, detailing its categorical distribution and token counts for both Chinese and English. The four sub-corpora include a total of 2,000 texts and more than four million tokens.

**Table 1:** Functional categories, registers, genres and tokens in the PCCE design.

Functional category	Register	Code	Genre	Texts	Tokens	
					Chinese	English
Informative	News	A-C	News	88	418,105	347,471
	General	D	Religious writing	17	67,254	68,432
		E	Skills, trade & hobbies	38	160,643	155,306
		F	Popular lore	44	186,283	198,815
		G	Biographies & essays	77	306,048	304,200
		H	Miscellaneous	30	136,645	126,970
	Academic	J	Science	80	346,431	321,970
Imaginative	Fiction	K	General fiction	29	125,436	119,409
		L	Mystery & detective	24	99,251	99,277
		M	Science fiction	6	24,186	22,303
		N	Adventure fiction	29	119,966	118,281
		P	Romantic fiction	29	121,853	121,420
		R	Humour	9	37,852	36,729
**Total**				500	2,149,953	2,040,583

### 3.2. Selection of linguistic predictors

The profound typological divergence between Chinese and English poses substantial methodological challenges for any joint analysis of source and target language data in studies investigating the shining-through effect across both Chinese-to-English and English-to-Chinese translational directions. The principal contribution of the present work resides in its systematic identification of linguistic indicators shared by both languages and in the development of a classification model designed to reliably detect the shining-through effect in bidirectional English–Chinese translation. As a prerequisite for understanding the rationale underlying the selection of these linguistic variables, it is necessary to first introduce several widely posited typological dichotomies between Chinese and English: the dynamic–stative, hypotactic–paratactic, compact–diffusive, passive–active and synthetic–analytic distinctions [48].

The dynamic–stative dichotomy pertains to the divergent preferences for verbal and nominal constructions in Chinese and English respectively [48, 104]. Whereas English exhibits a marked propensity for nominal expression, Chinese displays the converse tendency, with verbal constructions assuming a more prominent role. The dynamic character of Chinese is illustrated by the iterative deployment of verbs: in Example (1), the verb 去 (*qù*, ‘go’) is repeated to intensify the expression of movement. Example (2) demonstrates the phenomenon of verb reduplication in Chinese, a grammatical device that functions to attenuate the degree or intensity of the action denoted [49]. In this instance, the verbs 吃 (*chī*, ‘eat’) and 收拾 (*shōushi*, ‘tidy up’) are reduplicated as 吃吃 and 收拾收拾 respectively, conveying the sense that the action is performed in a mild or perfunctory manner. English, by contrast, exhibits a considerably greater reliance on nominal forms, a tendency most conspicuously manifested in the pervasive use of nominalisation [48, 105]. This is illustrated in Example (3), where the nominal forms *transparency* and *accountability* exemplify the characteristically nominal orientation of English expression.

The hypotactic–paratactic dichotomy concerns the differing ways in which Chinese and English achieve textual cohesion through the use of connective devices [48,49]. English characteristically relies on explicit grammatical mechanisms, such as conjunctions and subordinate clauses, to signal logical and syntactic relationships between and within sentences. Chinese, by contrast, frequently employs topic-chain structures as the primary means of organising discourse and maintaining cohesion. A particularly salient manifestation of this contrast is the prevalence of run-on sentences in Chinese, which occur even in formal academic writing, whereas equivalent constructions are generally regarded as grammatically inadmissible in English [50]. This is illustrated in Example (4), in which a single sentence encompasses seven syntagms, a degree of structural density that renders faithful single-sentence translation into grammatically well-formed English virtually impossible without the introduction of conjunctions.

The phenomenon of run-on sentences in Chinese also bears directly on the compact–diffusive dichotomy, which concerns the structural organisation of sentences across the two languages. English is characterised by logically sequenced and syntactically transparent sentence structures, whereas Chinese permits considerably greater structural flexibility and complexity [48]. This contrast is corroborated empirically by Jiang and Liu [51], whose comparative analysis demonstrated that Chinese sentences tended to exhibit greater mean dependency distances than their English counterparts. Given that run-on sentences are frequently analysed as a form of multi-layered compound sentence [50], their structural elaborateness is likely to exert pressure on translators to restructure sentences when rendering Chinese into English. Such restructuring may in turn affect the distribution of sentence-final punctuation marks, including full stops, question marks, semicolons and exclamation marks, as well as non-sentence-final punctuation, such as commas and colons, and may have broader consequences for the syntactic sophistication of the translated output [52]. In this connection, English translated from Chinese tended to display reduced levels of syntactic complexity relative to non-translated English, as reflected across multiple indices of sentence structure [12]. A further characteristic syntactic construction in Chinese is the existential sentence, illustrated in Example (5). Although the structurally analogous *there be* construction is also widely attested in English, existential sentences were found to occur with significantly greater frequency in original Chinese than in Chinese translated from English [52].

The passive-active dichotomy is a frequently discussed notion in Chinese-English contrastive analysis, highlighting a notable difference between Chinese and English in terms of voice preference. While passive constructions are less common in original Chinese texts, they are more frequently found in translated Chinese [16,17]. On the other hand, translated English texts from Chinese were found to use passive constructions less often [53].

Finally, the synthetic-analytic dichotomy reflects the difference between Chinese and English in the use of inflected forms to indicate grammatical relationships [1,48]. English shows variation in affixation, numbers, tenses and cases, etc., while in Chinese, these variations are rarely observed. For example, when expressing plural number, English uses plural forms, but Chinese tends to use classifiers, as indicated by 个 *ge* in 六个钟头 *liu ge zhongtou* ‘six hours’ in Example (2). This dichotomy can be also reflected by the use of modal verbs as English tends to rely on modal verbs to indicate modality, while the change in modality is often embedded in the context in Chinese [54].

(1) 我还是总得到那古园里去, 去它的老树下或荒草边或颓墙旁, 去默坐, 去呆想, 去推开耳边的嘈杂理一理纷乱的思绪, 去窥看自己的心魂.
  ‘I still often find myself going to that old garden, to sit under its ancient trees, by the overgrown grass, or beside the crumbling walls, sitting in silence, lost in thought, pushing aside the clamour in my ears to sort through my tangled thoughts, and peering into the depths of my own heart and soul.’
(2) 到那里恐怕已是十点多了, 吃吃饭, 收拾收拾东西, 我们只能有六个钟头休息的时间.
  ‘By the time we get there, it will probably be past ten o’clock. After having a meal and tidying up our things, we will only have six hours of rest time.’
(3) But she cautioned that the government needed to adopt international standards of transparency and accountability.
(4) ①父亲坐在书房里靠窗那堂软垫沙发上, ②两手捧着一盏新沏的铁观音, ③白烟袅袅, ④凄凄切切半蒙住他那张有风有霜的脸, ⑤沙发的蓝绒底子洒满翠绿竹叶, ⑥衬着窗外一丛幽篁, ⑦格外见出匠心.
  ‘①My father sat in the study, on the cushioned sofa by the window, ②holding a freshly brewed cup of Tieguanyin tea in both hands, ③the white smoke curled up, ④faintly veiling his weathered face, which had been touched by both wind and frost, ⑤the blue velvet base of the sofa was scattered with green bamboo leaves, ⑥complementing the cluster of dark bamboo outside the window, ⑦adding a special touch of craftsmanship.’
(5) 那声音有点新鲜.
  ‘The voice is a little new.’

The selection of linguistic indicators was guided by three criteria: theoretical relevance, cross-linguistic comparability and empirical interpretability. First, each feature was required to have a clear theoretical connection to one or more of the major typological distinctions between Chinese and English discussed above. Second, only features that could be operationalised in a broadly comparable manner across both languages were retained, ensuring that observed differences would reflect genuine linguistic variation rather than language-specific annotation artefacts. Third, the feature set was designed to capture lexical, syntactic and discourse-level dimensions of variation. On the basis of the typological distinctions outlined above, 34 linguistic variables were identified for the construction of the classification model, each of which is described in detail in Table 2.

**Table 2:** Linguistic indicators and descriptive statistics.

Dichotomy	Code	Mean	S.D.	Description
Dynamic-stative	noun	212.98	66.62	nouns
	verb	152.02	25.66	verbs
	adjective	47.41	28.84	adjectives
	adverb	48.62	15.83	adverbs
Hypotactic-paratactic	additive	19.76	11.61	additive connectives
	adversative	7.33	3.79	adversative connectives
	causal	10.09	7.83	causal connectives
	conditional	1.79	1.78	conditional connectives
	sequential	8.79	5.75	temporal connectives
	demonstrative	10.90	6.60	demonstrative pronouns
	first.person	11.80	14.69	first-person pronouns
	second.person	3.99	6.78	second-person pronoun
	third.person	26.35	21.55	third-person pronouns
Compact-diffusive	comma	60.20	21.29	commas
	period	43.04	13.52	periods
	exclamation	1.50	3.57	exclamation marks
	question	2.70	4.61	question marks
	semi.colon	2.47	3.38	semi-colons
	parenthesis	5.76	9.29	parentheses
	colon	3.24	4.50	colons
	ellipsis	0.65	2.56	ellipses
	dependency	3.97	0.93	mean dependency distance
	sentence.length	35.49	83.37	average sentence length
	existential	0.17	0.17	existential sentences
Passive-active	passive	0.09	0.09	passive constructions
Synthetic-analytic	unit	0.79	1.34	unit classifiers
	collective	2.00	2.45	collective classifiers
	container	0.13	0.50	container classifiers
	measure	0.85	2.00	standardised measures
	species	0.32	0.67	species classifiers
	arrangement	1.02	1.80	arrangement classifiers
	temporal	1.56	2.28	temporal classifiers
	verbal	0.73	0.98	verbal classifiers
	modal	9.97	5.76	modal verbs

With respect to parts of speech (POS), four categories of content words were incorporated into the analysis. Function words were excluded on the grounds that no consistent cross-linguistic correspondence exists between Chinese and English in this domain. Given that nouns and adjectives characteristically co-occur, as do verbs and adverbs [55], these four content word categories collectively serve as the primary means of operationalising the dynamic–stative dichotomy between the two languages. The second and third sets of features relate to cohesive devices, specifically connectives and pronouns. Although both demonstrative and personal pronouns are attested in Chinese and English, the two languages do not share an identical taxonomy of conjunctions. A cross-linguistic comparison yielded five conjunction categories common to both languages which were employed to examine the hypotactic–paratactic dichotomy. The compact–diffusive dichotomy was investigated through two further feature sets: punctuation marks and syntactic structures. Within the domain of syntax, passive constructions were subject to dedicated analysis as the primary means of operationalising the passive–active dichotomy. Mean dependency distance, average sentence length and existential sentences were included to operate compact-diffusive dichotomies due to their comparability across both languages. Finally, classifiers and modal verbs were incorporated as variables to probe the synthetic–analytic distinction. Several additional features were considered but ultimately excluded. For example, tense markers were not included because they partially overlap with verbal morphology and would introduce redundancy into the feature space. Likewise, language-specific features (e.g., *ba*-construction in Chinese, articles and cases in English) were excluded because they lacked clear equivalents in the other language, thereby reducing the validity of cross-linguistic comparisons.

A Python script was employed to extract the relevant variables, with frequency-based variables being normalised to a per-thousand-token scale. Before proceeding with the analysis, the dataset was subjected to a z-score transformation to standardise the numeric values, addressing disparities in their scales.

### 3.3. A semi-supervised multivariate approach

The multidimensionality of the dataset means that advanced multivariate techniques should be adopted for dimensionality-reduction analysis and interpretation of the results via visualisation. To facilitate the investigation of the typological differences between Chinese and English and the impact on their translations, a weakly-supervised multivariate approach proposed by Diwersy et al. [24] was applied in the present study. This approach based on the design of within-method triangulation is effective in revealing the interaction of different linguistic variables in shaping the complexity of translated languages [56].

Table 3 presents an overview of the seven-step analytical procedure underpinning this method. Broadly, the first three steps constitute the exploratory phase of the analysis, whilst the subsequent four steps are oriented toward confirmatory analysis by means of supervised machine learning techniques. The analytical workflow commences with the application of PCA to uncover latent dimensions that capture the distinctions of interest. As an unsupervised multivariate technique, PCA does not draw on categorical information from the dataset, with the consequence that variation attributable to extraneous factors is also represented in the resulting plot. Once the dimension associated with the linguistic category of interest has been identified, a more granular examination of that dimension is undertaken using a density plot, a smoothed analogue of a histogram, to render underlying distributional patterns visible. On the basis of this classification, the category of interest is subsequently designated as the response variable in a LDA in order to enhance classification accuracy. An analogous procedure is then applied to identify latent dimensions in which the target category is visually separable, with additional cross-validation performed using distinct training and testing datasets. To guard against circular reasoning and confirmatory bias, validation by means of a non-linear classifier, such as SVM, is further recommended. The interpretation of results is ultimately grounded in an evaluation of feature weights within the identified latent dimensions. For more detailed expositions of each procedural step, the reader is referred to Diwersy et al. [24] and Evert and Neumann [8].

**Table 3:** An overview of the weakly-supervised multivariate approach.

Stage	Step	Brief description
Exploratory	1	PCA is used to uncover the latent dimensions within the dataset, allowing for an exploration of its underlying structure;
	2	The PCA outcomes are visualised through scatterplot matrices to assess whether the linguistic categories of interest are effectively captured;
	3	If the desired dimensions are not identified, prior knowledge is utilised to refine the classification process and highlight specific textual categories;
Confirmatory	4	A supervised machine learning technique is employed to classify the linguistic categories of interest based on the dataset;
	5	The classification model is cross-validated. This step is further supported by the application of additional supervised machine learning methods to validate findings;
	6	The results of the supervised classification are also visualised using scatterplot matrices to confirm whether the linguistic categories of interest are identified;
	7	The feature weights that contribute to the identified latent dimensions are analysed to provide insights into the linguistic characteristics driving the observed patterns.

In the present study, a minor methodological modification was introduced: LDA was replaced by FDA in order to accommodate a violation of the multivariate normality assumption in the dataset (Henze-Zirkler test: HZ = 1.06, *p* < 0.001). First proposed by Hastie et al. [57], FDA constitutes a non-parametric extension of LDA that employs a non-linear regression framework to achieve superior classification performance under conditions in which the assumptions of multivariate normality and homogeneity of covariance matrices are not satisfied. Prior to model training, the dataset was randomly partitioned into a training set (80%) and a held-out test set (20%). FDA was implemented in R using the *fda* method in the *caret* package, which employs Multivariate Adaptive Regression Splines (MARS) as the underlying regression engine. Hyperparameter tuning was conducted on the training set using 10-fold cross-validation. A grid search was performed over the MARS parameters degree and nprune, and the optimal parameter combination (degree = 1, nprune = 44) was selected based on cross-validated classification accuracy. The final FDA model was then fitted using these parameters and evaluated on the held-out test set. While MARS provided more flexible modelling of non-linear predictor-response relationships, it does not yield directly interpretable coefficients for individual predictors. Therefore, a supplementary FDA model was fitted using polynomial regression as the basis function, which produces a single linear coefficient for each predictor on each discriminant dimension. This specification allows for a more straightforward characterisation of the direction and relative magnitude of each variable’s contribution to the discriminant space and was used exclusively for the purpose of model interpretation, rather than classification.

To quantify the degree of distributional similarity between variety pairs along the latent dimension, the overlap coefficient was computed using the overlapping package in R. This measure estimates the area of intersection between two kernel density estimates, ranging from 0 (no overlap) to 1 (complete overlap), thereby providing a continuous index of the extent to which two varieties occupy similar regions of the multivariate space.

## 4. Results and discussion

### 4.1. Exploratory analysis

**Fig 1:**
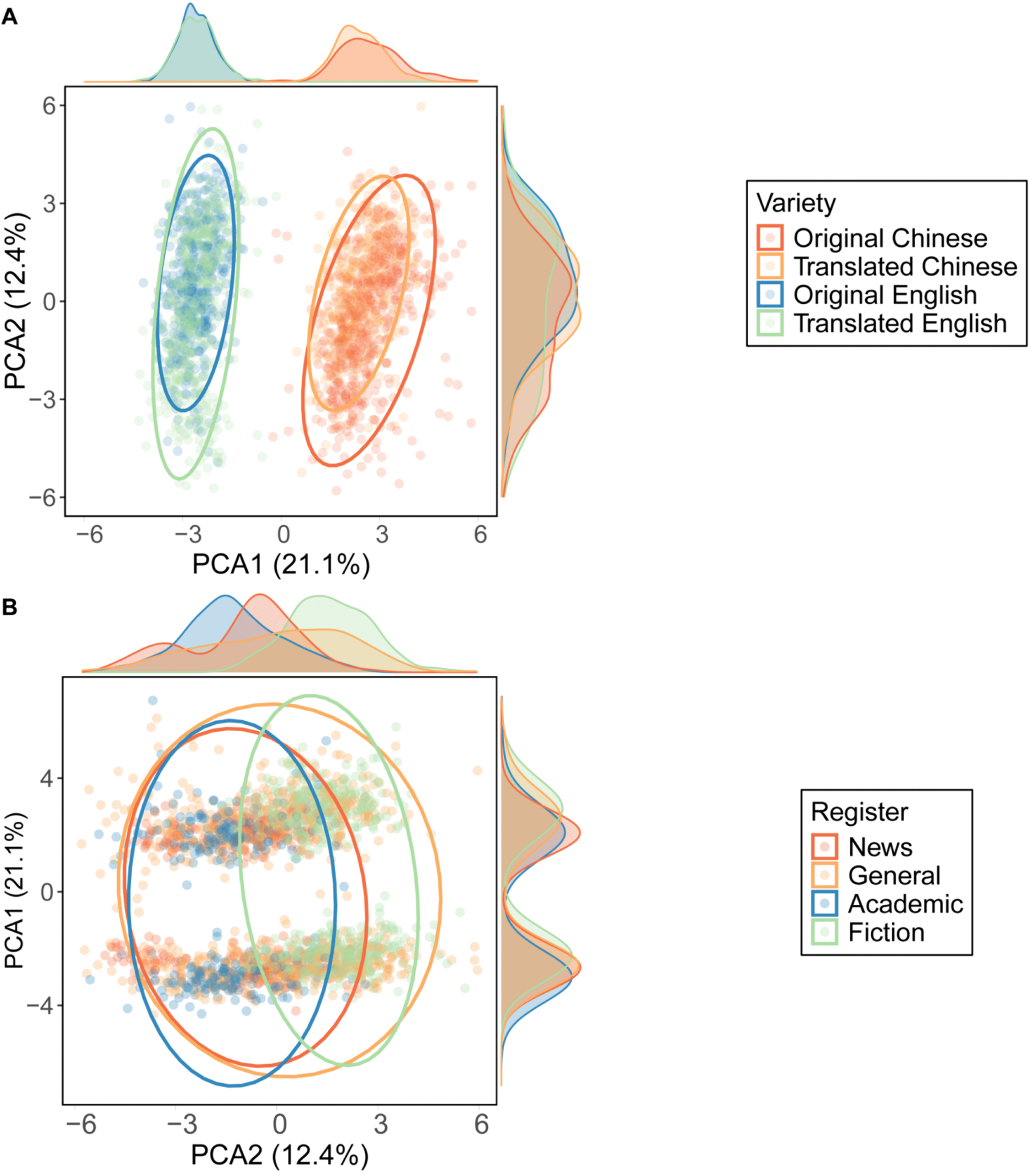
Biplots of the PCA results based on the first two dimensions and the density curves of dimension 1.

PCA was first applied to examine the structure of the entire dataset. Since categorical information was not incorporated into the analysis, the results potentially reflect both language varieties and registers. As illustrated, Fig 1 displays the intersection of the first two dimensions of the PCA, with dimension 1 (x-axis) explaining 21.1% of the variance and dimension 2 (y-axis) accounting for 12.4%. Along dimension 1 (Fig 1A), a clear separation between Chinese and English texts could be observed: both original and translated Chinese texts were positioned between 0 and +6, while original and translated English texts were located between −6 and 0. Examination of dimension 2 in Fig 1B seems to suggest that it was more closely associated with register variation. Specifically, journalistic and academic texts were predominantly situated in the negative coordinate range, whereas fictional texts were positioned in the opposite direction. General texts occupied a broader area, encompassing all three register categories in the biplot. On this basis, dimension 1 appears to correspond, at least in part, to the language-related distinction that is the focus of this study, although it should be noted that PCA dimensions reflect overall variance structure and may also be influenced by other, unmeasured sources of variation.

Additionally, some deviations are observed in the positioning of translated Chinese and English texts. This pattern is more clearly illustrated by the density curves along the x-axis above the biplot. The density plot, a smoothed histogram, depicts the distribution of different values in the dataset. It is also a useful tool for examining the relationship between variables. In this analysis, if two language varieties are similar, the density curves of their PCA coordinates should exhibit similar spatial positioning and configuration. From the density curves in Fig 1A, it can be observed that original Chinese and translated Chinese overlapped (overlap coefficient = 0.83), as did original English and translated English (overlap coefficient = 0.94). This suggests that translated texts might share similar linguistic features with the target languages. However, it is also evident that translated Chinese aligned more closely with original English (its source text) in terms of the two-peak and sharper shape. This observation is consistent with findings reported by Evert and Neumann [8] in their analysis of German and English translations. Moreover, translated English and Chinese texts were characterised by a slight tendency towards the middle, and this tendency was slightly more pronounced in translated Chinese.

**Fig 2:**
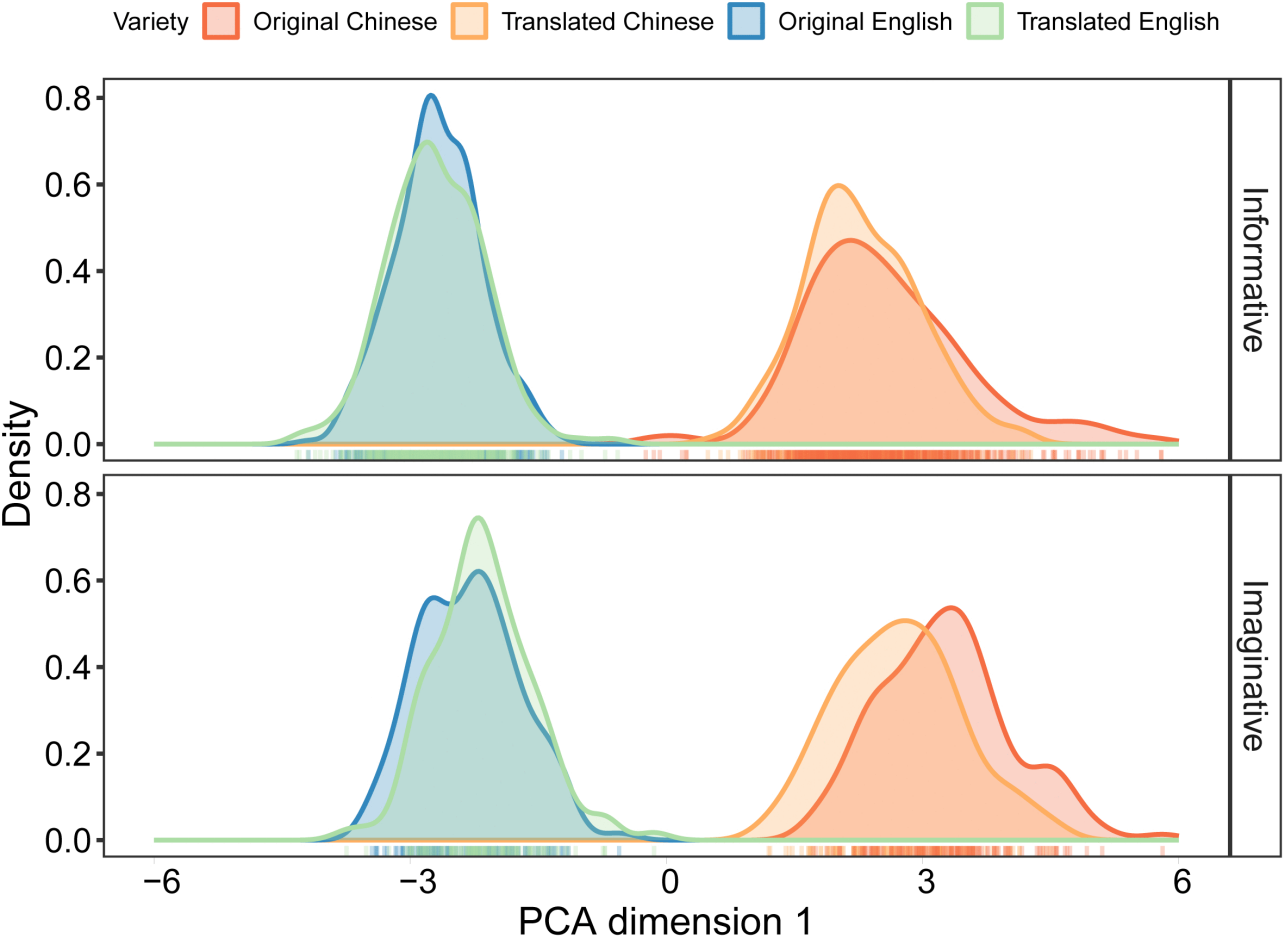
Density curves of PCA dimension 1 across four different registers.

Before proceeding to the confirmatory stage, the analysis is directed at the density plots of PCA dimension 1 across different registers. A closer examination of Fig 2 reveals a clear separation between English and Chinese, regardless of the registers. In informative texts, the positioning and shape of the translated texts did not closely resemble those of the original texts. However, in imaginative texts, a stronger tendency toward the source language could be observed particularly in translated Chinese. The overlap coefficient between translated Chinese and original Chinese was lower in imaginative texts (0.74) than in informative texts (0.86). These patterns may suggest a stronger shining-through effect in English-to-Chinese translations imaginative texts.

Overall, the PCA results provide preliminary evidence supporting the shining-through effect. However, several limitations of PCA should be noted. First, given that our analysis is based on a bidirectional parallel corpus (i.e., English-to-Chinese and Chinese-to-English), it remains unclear whether the observed pattern is attributable to the source text or the source language. Second, as PCA identifies variance structure rather than pre-specified linguistic categories, the observed separation along any given dimension may reflect typological differences between Chinese and English, source language shining through in translated texts, register variation or some combination of these factors. The interpretation of PCA dimensions in terms of theoretically meaningful constructs, i.e., the shining-through effect, should therefore be regarded as a tentative, post-hoc reading of the data rather than a direct empirical demonstration. As explained by Evert and Neumann [8], the PCA might collapse two or more dimensions into a single dimension due to its unsupervised nature. To address these concerns and gain a deeper understanding of the shining-through effect, it is essential to conduct a supervised multivariate analysis.

### 4.2. Confirmatory analysis

This section presents the results obtained from FDA. The model trained on the data achieved an accuracy rate of 85%, and a similar accuracy of approximately 85% was observed for the model’s predictions on the testing data. This accuracy is notably similar to the results reported by Evert and Neumann [8] and Baroni and Bernardini [58], indicating that our model is highly robust in distinguishing these four language varieties. Additionally, several other supervised machine learning models were built to provide a comprehensive evaluation of the reliability and validity of the selected variables, including SVM, random forest (RF), conditional inference tree (CIT), multinomial logistic regression (MLR). The performance of these models is summarised in Table 4, which reports the mean values of precision, sensitivity, specificity and F1 scores. As shown, the accuracy rates for all models are approximately 80%, but generally RF model reported the highest values in all evaluation metrics. Nevertheless, due to the advantages of FDA in terms of visualisation and data interpretation, we chose to proceed with the analysis based on the classification results obtained from FDA.

**Table 4:** Performance of machine learning models (mean values).

Model	Precision	Sensitivity	Specificity	F1 score
FDA	0.85	0.84	0.95	0.85
SVM	0.84	0.83	0.94	0.83
RF	0.89	0.89	0.96	0.89
CIT	0.75	0.74	0.91	0.74
MLR	0.83	0.82	0.94	0.82

**Fig 3:**
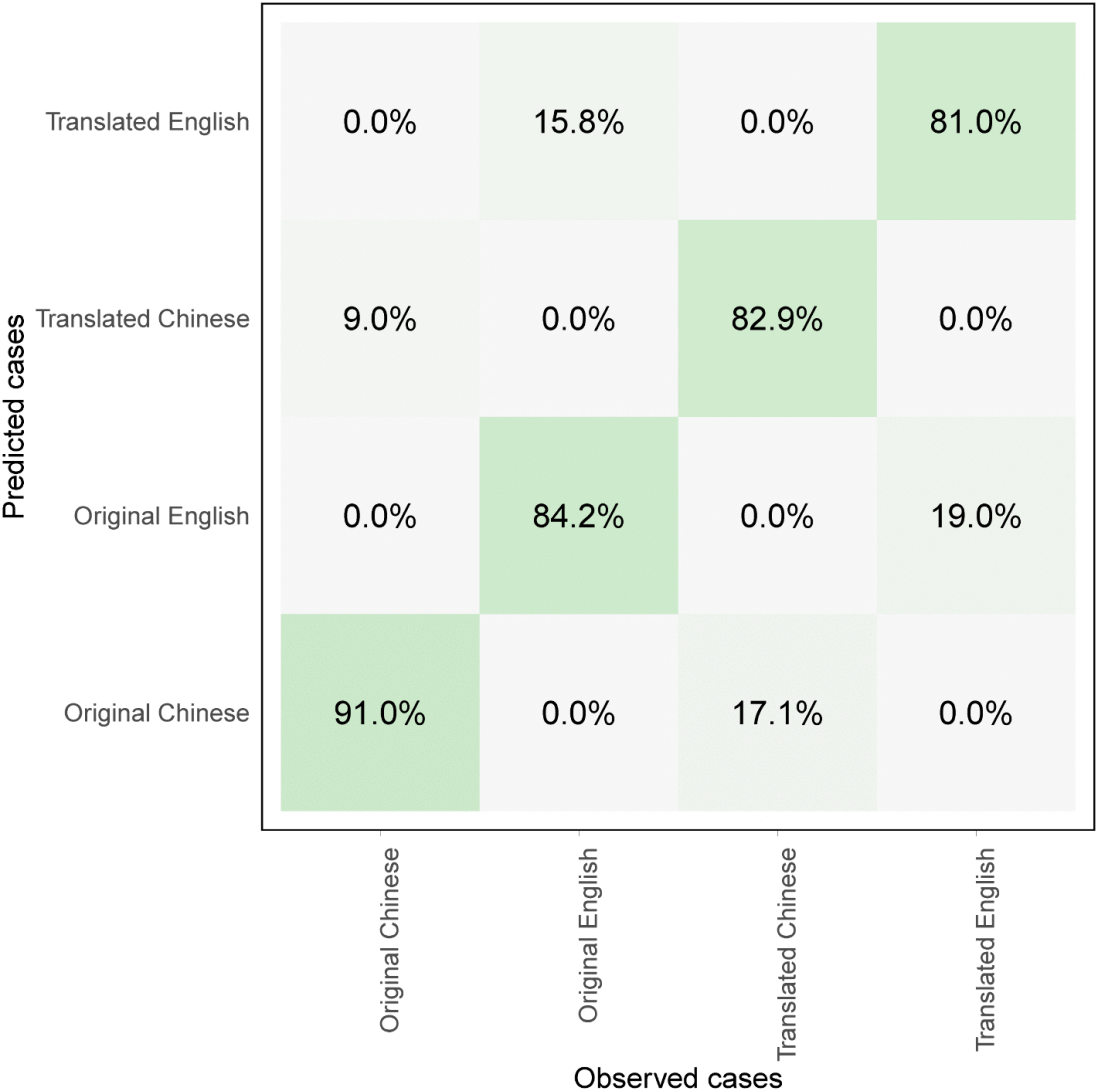
Confusion matrix of the FDA model based on testing data.

In addition to the examination of these metrics, an essential step in evaluating a machine learning model is the examination of the confusion matrix. This matrix provides insight into the model’s performance on each individual category. In the present study, the confusion matrix also highlights the relative similarity between language varieties. Specifically, if two language varieties are similar, the model may struggle to distinguish between them, leading to lower accuracy rates. Fig 3 presents the confusion matrix based on the testing data. The matrix consists of two axes: the horizontal axis represents the observed (true) cases in the dataset, while the vertical axis represents the predicted cases made by the model. As shown, the model performed exceptionally well in classifying original Chinese texts, with an accuracy rate of approximately 91.0%. The 9.0% of misclassifications occurred when original Chinese texts were incorrectly classified as translated Chinese. The model performed also better in classifying translated Chinese, with 82.9% of texts correctly identified, and the remaining 17.1% mistakenly classified as original Chinese. For the English varieties, the accuracy rate for classifying original English was around 84.2%, with roughly 15.8% of texts misclassified as translated English. A similar accuracy rate was observed in the classification of translated English (81.0%), and 19.0% of texts were misidentified as original English. The confusion matrix further reveals that no Chinese texts were misclassified as English, which underscores the high validity and reliability of the model in selecting relevant variables for the contrastive analysis of typological differences between Chinese and English. Additionally, translated texts were more easily confused with their target languages in both Chinese and English, potentially suggesting the presence of normalisation effect. Finally, translated Chinese was more readily identified than translated English, possibly reflecting the stronger influence of the shining-through effect. This pattern may also indicate the impact of language prestige, as the translation direction from a dominant to a minor language could amplify this effect.

**Fig 4:**
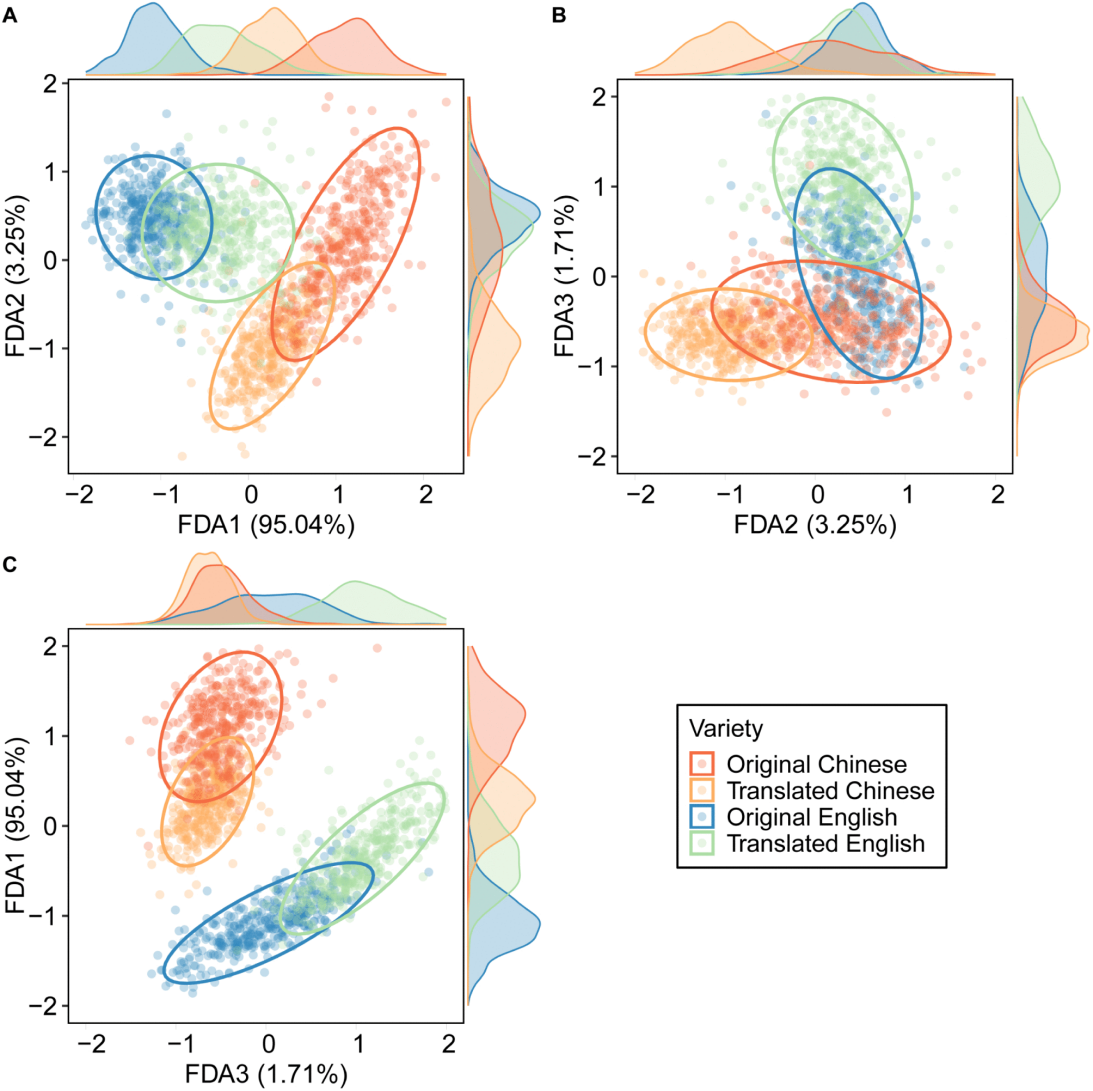
Biplots of the three dimensions generated by the FDA model.

A similar procedure was applied to interpret the visualised outcomes of the FDA model. The algorithm identified three latent dimensions, which explain 95.04%, 3.25% and 1.71% of the variance, respectively. Fig 4A presents the biplots for the first two dimensions, along with the corresponding density curves. Biplots and density curves for the other dimensions are provided in Figs 4B and 4C. Overall, the four language varieties were largely distinguishable, with English texts predominantly located in the negative coordinate range and Chinese texts positioned in the positive coordinate range. A closer inspection of the density plot for dimension 1 reveals that both translated varieties were displaced from their respective originals toward a more central position. Wilcoxon Rank Sum tests confirmed that these displacements were statistically significant for both English-to-Chinese (*z* = 21.8, *p* < 0.001) and Chinese-to-English translations (*z* = −21.1, *p* < 0.001). To further examine the degree of distributional similarity between translated and original texts, kernel density overlap coefficients were computed: a lower overlap value indicates greater distributional divergence between a translated variety and its corresponding original, which may be interpreted as reflecting a stronger shining-through effect. English-to-Chinese translations showed an overlap of 0.26 with Chinese originals, compared to 0.31 for Chinese-to-English translations with English originals, suggesting that English-to-Chinese translations were distributionally more distant from their target-language baseline. This pattern may suggest a stronger shining-through effect in English-to-Chinese translations.

It is important to note that Figs 4B and 4C highlight additional translationese effects, distinguishing translated Chinese and English texts from other language varieties. Specifically, Fig 4B shows that translated Chinese texts were more distinctly separated from the other language varieties, while Fig 4C illustrates that English translations deviated from other texts. Notably, the deviation of translated Chinese texts was more pronounced than that of translated English texts. This finding aligns with the results from the confusion matrix, which indicated that translated Chinese texts were more easily identifiable than their English counterparts.

Although the analysis above has indicated a stronger tendency toward the shining-through effect in the English-to-Chinese translation direction compared to the reverse direction, it remains crucial to determine whether this observed tendency is driven by the source language rather than the source text itself. To address this issue, a correlation analysis was recommended to assess whether the FDA or PCA coordinates of the source text are correlated with those of its translation [8]. If a strong correlation is found between the source text and its translation (*r* > 0.7), the observed shining-through effect should be interpreted as interference from the source text (e.g., author style, topic domain or relevant linguistic features). On the other hand, if no strong correlation is found, but the visual analysis reveals that translated texts exhibit characteristics more aligned with the source language, this tendency should be identified as the “genuine” shining-through effect, that is, the source language influencing the translation.

**Fig 5:**
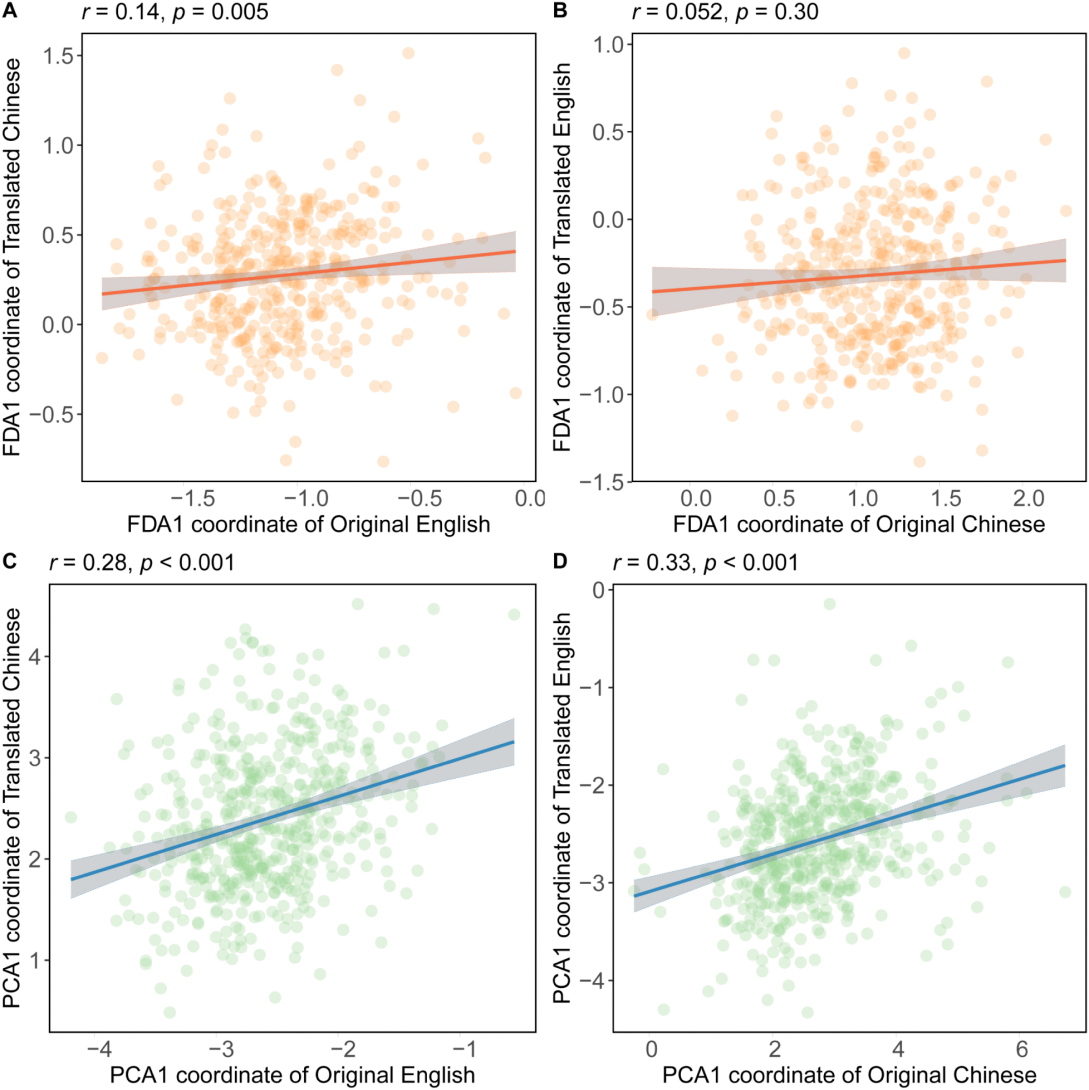
Spearman correlation analysis of the original texts and their translations in FDA and PCA scores.

To further substantiate our findings, a Spearman correlation analysis was conducted on the FDA and PCA scores of the original texts and their translations. Fig 5 presents the scatterplots of the correlation analysis. In each of the four scatterplots, the horizontal axis corresponds to the scores of the original texts, while the vertical axis represents the scores of the translated texts, as derived from the FDA and PCA dimensions that potentially reflect the shining-through effect. Figs 5A and 5B reveal no strong correlation between the FDA scores of the originals and those of their translations (*r* < 0.2). However, Fig 5C shows that the PCA scores of English originals were significantly correlated with the scores of their Chinese translations (*r* = 0.28, *p* < 0.01), though the correlation was not very strong. A similar trend is observed in Fig 5D, where the correlation was slightly stronger (*r* = 0.33, *p* < 0.001). Based on these correlation results, we may conclude that the shining-through effect was more effectively captured by the FDA model than by the PCA model.

**Fig 6:**
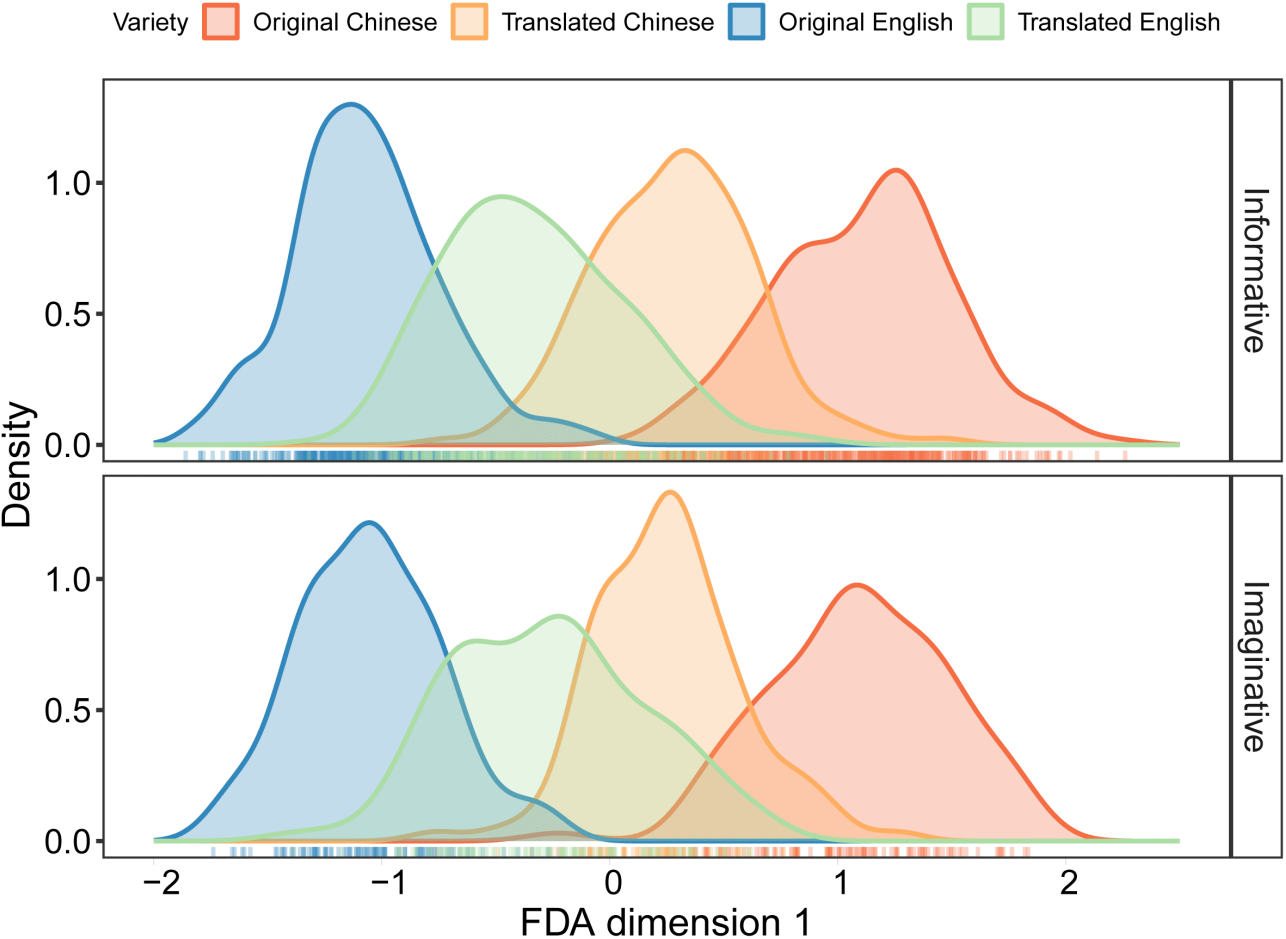
Density plots of the FDA dimension 1 across four different registers.

Having confirmed this, we proceeded with further analysis by examining the distribution density of dimension 1 from the FDA across four distinct registers. Fig 6 presents the density plots for these four language varieties within informative and imaginative texts. It is clear that the translated texts were consistently positioned between the original Chinese and English texts. However, some variability was observed across two categories of text functions. Comparing the position of the four language varieties, it appears that in the category of imaginative texts, translated English and Chinese exhibited a stronger tendency towards the middle position than in informative texts. Furthermore, in the translation direction from English to Chinese, translated imaginative texts (overlapping coefficient = 0.26) displayed a slightly greater deviation from original Chinese than translated informative texts (overlapping coefficient = 0.27). These patterns suggest that translations of imaginative texts might display a more pronounced tendency towards source language shining through than translations of informative texts, and that the prestige effect seemed to be slightly more visible in imaginative texts than informative texts.

### 4.3. Feature importance analysis

**Fig 7:**
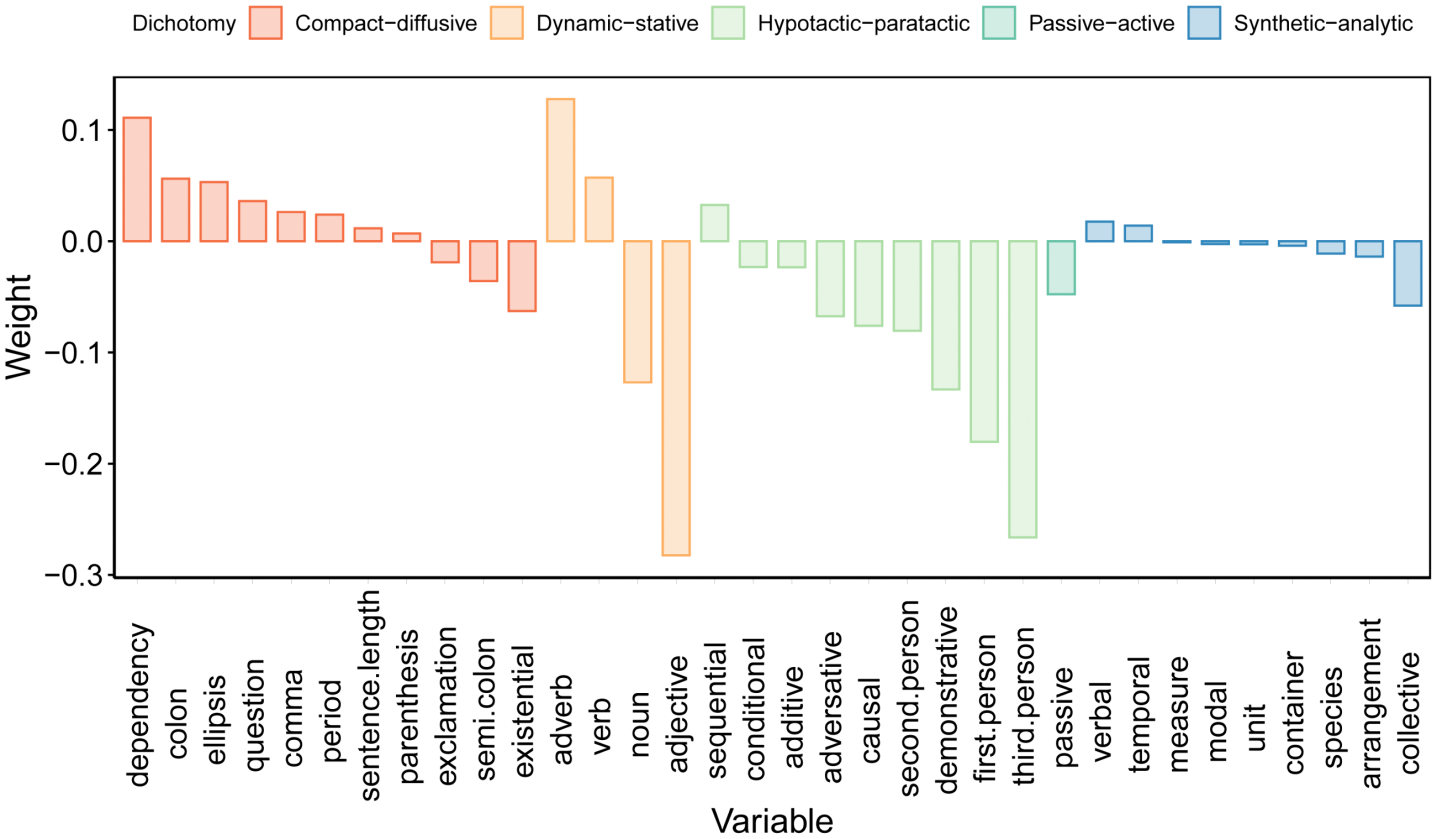
Feature weights contributing to the dimension 1 of the FDA model.

With the previous analyses completed, we proceeded to the final step of the procedure: examining the feature weights contributing to the dimension that yielded the best classification results. Fig 7 illustrates the weights of the variables that contribute to the first dimension of the FDA model. As a reminder, English was positioned in the negative coordinate range, while Chinese was situated on the positive side of the horizontal axis in dimension 1. Variables with positive weights are associated with original Chinese, while negative weights correspond to features characteristic of original English. Notably, POS category emerged as some of the most significant contributing features. This suggests that the dynamic-stative dichotomy represented one of the most fundamental typological differences between Chinese and English in our dataset. The second most significant difference between these languages appeared to be related to the hypotactic-paratactic dichotomy. Pronouns and conjunctions, particularly third-person pronouns and causal connectives, were also crucial for the classification model. Additionally, passive constructions also emerged as one of the most important syntactic variables, indicating the presence of a passive-active dichotomy in the typological contrast between Chinese and English. It is worth noting that mean dependency distance was the most influential variable within the compact-diffusive category. Lastly, classifiers and modal verbs were comparatively less important based on the feature weights in dimension 1.

**Fig 8:**
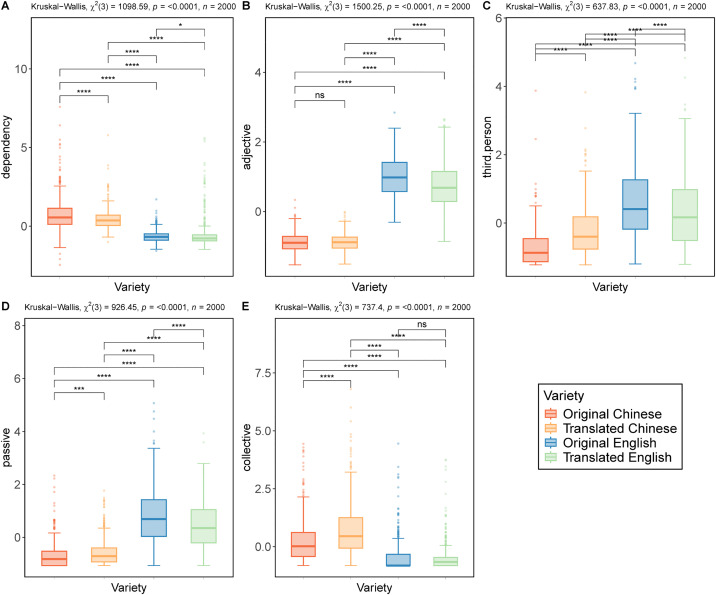
Univariate analysis of the key variables in each dichotomy.

A detailed comparison of the key variables contributing to the typological differences between Chinese and English is presented in Fig 8. The Kruskal-Wallis test was employed as an omnibus test to assess overall group differences across the four varieties. Pairwise post-hoc comparisons were subsequently conducted using the Wilcoxon Rank Sum test for independent samples (e.g., original Chinese vs original English) and the Wilcoxon Signed-Rank test for paired samples (e.g., original Chinese vs translated English, and original English vs translated Chinese). Due to space limitations, not all variables are analysed; instead, only those variables that contribute significantly to the typological differences between the two languages are further examined.

Fig 8A demonstrates a significant difference between Chinese and English in mean dependency distance (*ꭓ²* = 1098.59, *df* = 3, *p* < 0.001), with Chinese texts having higher values than English texts (*z* = 24.8, *p* < 0.001), and it also shows that original Chinese texts were characterised by a significantly longer distance than translated Chinese texts (*z* = 5.37, *p* < 0.001). Fig 8B presents the significant differences in the use of adjectives (*ꭓ²* = 1500.25, *df* = 3, *p* < 0.001), revealing that adjectives were used significantly more frequently in English than Chinese (*z* = 27.3, *p* < 0.001). The shining-through effect seems to be evident in this feature, as translated English from Chinese used adjectives significantly less frequently than original English texts (*z* = −6.74, *p* < 0.001). Fig 8C further corroborates the hypotactic-paratactic dichotomy, showing that third-person pronouns were significantly less frequently employed in both translated and original Chinese texts (*ꭓ²* = 637.83, *df* = 3, *p* < 0.001). This indicator also demonstrates the shining-through effect, as evidenced by a significantly higher frequency of third-person pronouns in translated Chinese than in original Chinese (*z* = 12.8, *p* < 0.001), and a significantly lower frequency in translated English than in original English (*z* = −4.73, *p* < 0.001). Fig 8D illustrates the significant differences in using passive constructions across four varieties (*ꭓ²* = 926.45, *df* = 3, *p* < 0.001). Passive constructions were significantly underrepresented in translated English texts (*z* = −5.05, *p* < 0.001) but slightly overused in translated Chinese texts (*z* = 4.03, *p* < 0.001). This suggests that the shining-through effect was observable in this linguistic feature. Lastly, Fig 8E indicates significant differences in the frequency of collective classifiers (*ꭓ²* = 737.4, *df* = 3, *p* < 0.001), with a significantly higher frequency of collective classifiers observed in Chinese than in English texts (z = 15.5, *p* < 0.001). In summary, the analysis of these selected variables highlights the typological differences between Chinese and English, while also demonstrating how these differences contributed to the manifestation of source language shining through in both translation directions.

## 5. Discussion

In this section, the three research questions are revisited and addressed through the findings derived from the PCA and FDA analyses. Overall, the model constructed using 34 linguistic variables shared by Chinese and English achieved a high level of classification performance and provided a useful basis for exploring patterns associated with typological differences and source language shining through via dimensionality-reduction and visualisation techniques.

For the first research question, our analysis provides empirical evidence for the presence of the source language shining through effect in translations from Chinese to English and English to Chinese. The PCA results clearly separated Chinese and English along the first principal component, which accounts for the largest proportion of variance. The density plot for the first PCA dimension may indicate that both translated Chinese and English texts showed more normalisation effects, as evidenced by the position relative to the original texts. Although correlation analysis suggests that some of this similarity might stem from source text interference, subsequent FDA analysis offers further empirical support for the existence of “genuine” source language shining through. The confusion matrix generated by the FDA model reveals that no English texts were misclassified as Chinese, and the overall classification accuracy was satisfactorily high. This result was further corroborated by the FDA visualisations, where Chinese and English texts were distinctly separated along the first dimension. In the density plot of the first FDA dimension, translated Chinese and English texts occupied positions intermediate between source-language and target-language varieties, a pattern that is consistent with the presence of source language shining through in both translation directions. The univariate analysis of several key variables also provided evidence for the typological differences between these two languages and the shining-through effects in both directions. For example, the longer mean dependency distance in Chinese than English aligns with the finding of Jiang and Liu [51], and the overrepresentation of passive constructions and third person pronouns in translated Chinese is in line with the findings of previous studies [16–18,52]. When comparing the variance explained by the dimension capturing the shining-through effect in discriminant analysis, our findings reveal that the first dimension accounts for over 90% of the variance. This is substantially higher than the approximately 6.5% reported by Evert and Neumann [8], who studied German-English bidirectional translations. These results seem to offer further empirical support for the typological difference hypothesis, aligning with previous studies [27–30].

The results of the FDA analysis also provide insights into the second research question. In the first FDA dimension, it is evident that translated Chinese from English exhibited a more pronounced tendency toward the original English than the reverse translation direction. This finding aligns with the language prestige hypothesis in typologically distinct languages and is consistent with previous studies [8,26]. However, a comparison with the findings of Evert and Neumann [8] suggests a potentially interesting contrast: whereas the language prestige effect might be expected to produce an even stronger shining-through effect in English-to-Chinese translation than that observed for the typologically closer English-German pair, the present results do not indicate a comparatively stronger effect for Chinese. One possible, and admittedly speculative, interpretation is that the substantial typological distance between Chinese and English may attenuate the prestige effect to some extent, such that typological distance could act as a moderating factor in the relationship between language prestige and shining-through. More specifically, in the translation direction from a high-status language to a low-status one, the prestige effect is more likely to manifest when the source and target languages are typologically similar. However, when the source and target languages are typologically different, the prestige effect is likely to be relatively diminished. It should be emphasised, however, that this moderation is not directly tested in the present study. The present design does not include a typologically closer language pair for comparison within the same analytical framework, and the observation is based on a comparison across studies that differ in corpora, operationalisations and methods. As such, this interpretation should be regarded as a theoretically motivated hypothesis for future research, rather than as direct empirical evidence of a moderating effect.

Finally, our analysis demonstrates that the shining-through effect is consistently observable across different registers, albeit with varying degrees of intensity across registers. The consistent presence of this effect across both translation directions and four registers in the present dataset suggests that source language shining through may be a pervasive, if not universal, tendency within the Chinese-English language pair as represented in the PCCE corpus, though whether this extends to other corpora or language pairs remains an open empirical question. However, in line with the previous findings [11,14,21], register variation emerged as a critical factor influencing the strength of the shining-through effect. Our analysis indicates that within the dataset, translated imaginative texts exhibited a stronger tendency toward shining-through effect than informative texts. This variability in the strength of the shining-through effect provides further support for the risk-aversion hypothesis [31–33]. These patterns are consistent with the possibility that translators may adapt their linguistic strategies according to target-culture norms and audience expectations, as shown in many other corpus-based studies [11,21,22]. The data from this study suggest that fictional translations tended to retain more source language features, which may indicate a greater inclination among translators to preserve source language characteristics in this register. Conversely, journalistic, general and academic translations appeared more target-language oriented, a pattern that could reflect the adoption of a standardisation strategy. Upon examining the genre composition of journalistic, general and academic texts, it becomes apparent that these texts were typically characterised by relatively specialised and restricted readerships. As a result, they might present greater risks for translators, which in turn possibly encouraged the adoption of a more conservative translation strategy.

## 6. Conclusion

The present study proposed a classification model to detect the shining-through effect in English-Chinese bidirectional translations by applying two multivariate techniques to 34 linguistic variables that can be both measured in Chinese and English. Through the combination of the PCA and FDA, the analysis revealed a stronger tendency towards source language shining through in the translated texts from English to Chinese than the opposite direction. Furthermore, the observed shining-through effect varied across different registers, with fictional texts showing a more pronounced tendency than other texts.

These findings have important implications for both the study of translation universals and translation practice. Firstly, our analysis further substantiates the notion that source language shining through is a robust and universal feature of translated language. This phenomenon has been observed consistently across both translation directions and registers. Furthermore, while this study provides new evidence supporting the language prestige hypothesis and risk-aversion hypothesis, it reveals the moderating role of typological distance in translations from a more prestigious to a less prestigious language. These insights deepen our understanding of the interplay between linguistic, social and cognitive factors in shaping the characteristics of translated texts. Finally, the linguistic strategies identified across different registers may serve as valuable guidelines for translators working with texts intended for specific purposes, particularly in terms of minimising communicative risks.

Several limitations of the present study should be acknowledged to guide future research. Firstly, while this study provides new evidence broadly consistent with the language prestige hypothesis and the risk-aversion hypothesis, it also raises the possibility, based on comparisons with previous studies involving typologically closer language pairs, that typological distance may influence the extent to which language prestige is reflected in source language shining through. Because this possibility was not directly tested in the present study, it should be regarded as a theoretical interpretation rather than an empirically established finding. Nevertheless, it points to a potentially fruitful direction for future research on the interaction between linguistic and sociolinguistic factors in translation. Secondly, although the corpus includes multiple registers, the observed patterns may still be partly influenced by genre-specific characteristics associated with the particular text types represented in the dataset. The extent to which these findings generalise to other genres therefore remains to be examined. Thirdly, the present corpus-based design does not allow for a systematic investigation of translator variability. Individual differences in translation experience, stylistic preferences and decision-making strategies may contribute to variation in source language shining through and warrant further study. Furthermore, additional research is needed to validate the findings in other Chinese–English comparable corpora beyond PCCE and across a wider range of typologically distant language pairs. Finally, incorporating a broader range of explanatory variables may provide a more comprehensive understanding of source language shining through. As noted by De Sutter and Lefer [20], linguistic choices in translation are shaped by multiple interacting factors, including temporality, spatiality, translator-related variables, and technological developments. Such a multifactorial perspective may offer valuable insights into both diachronic and synchronic variation in translated language.
