# Correction: Does language prestige hypothesis hold true across typologically distant languages? A corpus-based study comparing shining-through effect in English-Chinese bidirectional translations

**DOI:** 10.1371/journal.pone.0356799

**Published:** 2026-08-20

**Authors:** Jia Li, Yunhao Pan

The hyperlink in the Data Availability statement is incorrect. The correct hyperlink is: https://doi.org/10.17605/OSF.IO/BAU9W.

In the Introduction section, there is an error in the second sentence of the first paragraph. The correct sentence is: It was Baker [3], however, who introduced the more systematic notion of translation universals to denote those linguistic properties considered intrinsic to translated language.

In the Language prestige hypothesis subsection of the Related work, there is an error in the second sentence of the first paragraph. The correct sentence is: In his formulation of the two laws (the “law of interference” and the “law of growing standardisation”), Toury [25] describes how textual features from the source language are transferred or adapted to the target language.

In the Language prestige hypothesis subsection of the Related work, there is an error in the fourth sentence of the first paragraph. The correct sentence is: In this context, Toury [25] also introduced the language prestige hypothesis: when the text is translated from a less dominant language to a more prestigious one, the translation tends to be standardised, with less interference from the source text; however, when the text is translated from a dominant language to a minor one, the translation is more likely to show interference.

In the Present study subsection of the Related work, there is an error in the fourth sentence of the fourth paragraph. The correct sentence is: Crystal [39] defines a global language as one that holds a “special role that is recognised in every country”, while Ammon [40] further distinguishes between a language’s global status (geographical coverage), global function (use in international communication) and the factors influencing that function (the sociopolitical and economic motivations for its global use).

In the Present study subsection of the Related work, there is an error in the sixth sentence of the fourth paragraph. The correct sentence is: As McArthur [43] notes, “the Chinese language complex is much larger than that of native English users, but its members are largely ethnically and culturally homogeneous, and its worldwide distribution is limited”.

In the Selection of linguistic predictors subsection of the Data and methods, there is an error in the first sentence of the second paragraph. The correct sentence is: The dynamic–stative dichotomy pertains to the divergent preferences for verbal and nominal constructions in Chinese and English respectively [48].

In the Selection of linguistic predictors subsection of the Data and methods, there is an error in the sixth sentence of the second paragraph. The correct sentence is: English, by contrast, exhibits a considerably greater reliance on nominal forms, a tendency most conspicuously manifested in the pervasive use of nominalisation [48].

The subheading ‘3.1 Corpus’ of the Data and Methods should have been written as ‘3.1 Corpus design’.

In the Selection of linguistic predictors subsection of the Data and Methods, there is an error in the third sentence of the sixth paragraph. The correct sentence is: For example, when expressing plural number, English uses plural forms, but Chinese tends to use classifiers, as indicated by 个 ge in 六个钟头 (liù gè zhōngtóu, ‘six hours’) in Example (2).

In the Confirmatory analysis subsection of the Results and discussion, there is an error in the fifth sentence of the sixth paragraph. The correct sentence is: However, Fig 5C shows that the PCA scores of English originals were significantly correlated with the scores of their Chinese translations (r = 0.28, p < 0.001), though the correlation was not very strong.
