# Supplementary material for: Does language prestige hypothesis hold true across typologically distant languages? A corpus-based study comparing shining-through effect in English-Chinese bidirectional translations
Source: PLoS One. 2026 Jul 22;21(7):e0353687. doi: 10.1371/journal.pone.0353687 (PMC13390861; doi:10.1371/journal.pone.0353687)
Supplement: Attachment — Submitted filename: Response to Reviewers.docx [file pone.0353687.s002.docx]

**Response Letter to the Editors’ and Reviewers’ Comments**

**Date:** 23 June 2026

**Manuscript Number:** PONE-D-26-21657

**Manuscript Title:** Does language prestige hypothesis hold true across typologically distant languages? A corpus-based study comparing shining-through effect in English-Chinese bidirectional translations

Dear Editors and Reviewers,

We would like to express our sincere gratitude for the opportunity to submit a revised version of our manuscript titled “Does language prestige hypothesis hold true across typologically distant languages? A corpus-based study comparing shining-through effect in English-Chinese bidirectional translations” to *PLOS ONE*. We are deeply grateful for the time and effort invested by you and the reviewers in providing thoughtful feedback and constructive suggestions. These have been instrumental in helping us refine and strengthen our work.

In response to the reviewers’ suggestions and editorial guidance, we have made the following key revisions:

a. Strengthened the theoretical framing by clarifying the conceptualisation of key constructs, including language prestige and typological distance;
b. Enhanced methodological transparency and reproducibility by providing more detailed descriptions of feature selection, data preprocessing, FDA implementation, hyperparameter tuning, cross-validation procedures, model validation and statistical reporting;
c. Improved the interpretation of multivariate analyses by distinguishing more explicitly between statistical patterns and theoretical explanations;
d. Expanded the discussion of limitations and scope conditions.

We believe that these revisions have substantially improved the clarity, methodological rigor, theoretical precision and transparency of the manuscript. The revised version provides a more explicit account of the analytical procedures, a more carefully qualified interpretation of the findings, and a clearer distinction between empirical evidence and theoretical inference. As a result, the study offers a more robust and nuanced contribution to corpus-based translation studies and to ongoing discussions concerning source language shining through, language prestige and typological differences in translation. We hope that the revised manuscript now meets the expectations of the journal and we sincerely appreciate your consideration.

In accordance with the instructions outlined in your letter dated 6 June 2026, we have uploaded both a clean version of the revised manuscript and a marked-up version with all changes highlighted in dark blue using MS Word.

Please find enclosed our point-by-point response to the editors’ and reviewers’ comments. Each comment is numbered and reproduced in *italicised* red font. Our responses follow immediately in purple font, with corresponding excerpts from the revised manuscript presented in dark blue.

Thank you once again for your valuable feedback and continued consideration of our work.

Yours sincerely,

Author(s)

___________________________________________________________________________________

**Comments from the Editorial Office**

Comment 1

*Please ensure that your manuscript meets PLOS ONE’s style requirements, including those for file naming.*

**Response:**

Thanks for highlighting this issue. We have carefully reviewed the PLOS ONE style templates provided and revised the manuscript accordingly to ensure full compliance with the journal’s formatting requirements, including file naming conventions, heading styles and reference formatting.

Comment 2

*Please note that PLOS One has specific guidelines on code sharing for submissions in which author-generated code underpins the findings in the manuscript. In these cases, we expect all author-generated code to be made available without restrictions upon publication of the work.*

**Response:**

Thanks for pointing out this issue. We have reviewed the PLOS ONE guidelines on code sharing. All author-generated R code underlying the analyses reported in this manuscript has been made publicly available without restrictions. The code has been deposited at OSF: [https://doi.org/10.7910/DVN/205YXZ](https://osf.io/bau9w/overview?view_only=4d0810d7a1f54e6481d10e44e5711556) and is fully documented to facilitate reproducibility and reuse. A reference to this repository has been included in the section of data availability revised manuscript. Please note that the repository link provided is currently restricted to peer-review access and will be replaced with a publicly accessible version upon acceptance.

Comment 3

*Please ensure that you include a title page within your main document. You should list all authors and all affiliations as per our author instructions and clearly indicate the corresponding author.*

**Response:**

Thanks for this comment. A title page has been included within the main document. It lists all authors, their respective affiliations and clearly indicates the corresponding author, in accordance with PLOS ONE author instructions.

Comment 4

*In the online submission form you indicate that your data is not available for proprietary reasons and have provided a contact point for accessing this data. Please note that your current contact point is a co-author on this manuscript. According to our Data Policy, the contact point must not be an author on the manuscript and must be an institutional contact, ideally not an individual. Please revise your data statement to a non-author institutional point of contact, such as a data access or ethics committee, and send this to us via return email. Please also include contact information for the third party organization, and please include the full citation of where the data can be found.*

**Response:**

We thank the editorial team for drawing our attention to this issue. We acknowledge that the original contact point listed was a co-author, which does not comply with PLOS ONE’s Data Policy. We have revised the Data Availability Statement accordingly. The data underlying this study are openly accessible in the OSF repository. Below is the revised Data availability statement:

The datasets generated by the research during and/or analysed during the current study are available in the OSF repository, [https://doi.org/10.7910/DVN/205YXZ](https://osf.io/bau9w/overview?view_only=4d0810d7a1f54e6481d10e44e5711556)

Please note that the repository link provided is currently restricted to peer-review access and will be replaced with a publicly accessible version upon acceptance.

Comment 5

*We notice that your supplementary figures are uploaded with the file type ‘Figure’. Please amend the file type to ‘Supporting Information’. Please ensure that each Supporting Information file has a legend listed in the manuscript after the references list.*

**Response:**

Thank you for pointing out this issue. Upon reflection, we have decided to incorporate the supplementary figures directly into the main text as numbered figures, rather than retaining them as supporting information files. The figures have been repositioned and renumbered accordingly within the manuscript, and the corresponding in-text citations and captions have been updated to reflect this change.

___________________________________________________________________________________

**Comments from Reviewer #1:**

*The manuscript addresses an important and theoretically interesting question concerning the interaction of language prestige, typological distance, and source-language shining-through in English-Chinese bidirectional translation. The use of a bidirectional parallel corpus and a multivariate analytical framework represents a valuable contribution to translation studies. The study presents substantial evidence that shining-through effects occur in both translation directions and that these effects vary across registers. The corpus design and the effort to identify cross-linguistically comparable features are commendable.* *However, several important issues require clarification and revision before the manuscript can be considered for publication.*

**Response:**

We would like to express our sincere gratitude for the time and effort you dedicated to evaluating the initial version of our manuscript. We are deeply appreciative of your thorough and professional review, as well as your insightful suggestions for improvement, with which we fully agree. In light of your comments, we have substantially revised the manuscript to enhance its conceptual grounding, methodological transparency and analytical precision. The main revisions and additions are summarised as follows:

a. Strengthened the theoretical framing by clarifying the conceptualisation of key constructs, including language prestige and source language shining through, and by carefully qualifying claims concerning typological distance, translation universals and causal interpretations.
b. Enhanced methodological transparency and reproducibility by providing more detailed descriptions of feature selection, data preprocessing, FDA implementation, hyperparameter tuning, cross-validation procedures, model validation and statistical reporting.
c. Improved the interpretation of multivariate analyses by distinguishing more explicitly between statistical patterns and theoretical explanations.
d. Expanded the discussion of limitations and scope conditions, including translator variability, register-specific effects, corpus-related constraints and the generalisability of the findings beyond the PCCE corpus and the English–Chinese language pair.

Concerning your specific comments, we have numbered and quoted them in *italicised* red font. Our responses follow each comment in purple font, with modifications and additions from the manuscript presented in dark blue font below our responses.

Comment 1:

*The most significant concern is the claim that typological distance moderates the relationship between language prestige and shining-through. This moderation effect is not directly tested; instead, the argument is inferred through comparison with previous studies involving different language pairs. At present, the findings demonstrate directional differences in shining-through effects, but they do not permit a direct test of whether typological distance moderates the relationship between language prestige and source-language influence. The manuscript should either provide stronger evidence supporting this moderation claim or substantially soften and reframe it as a theoretical interpretation.*

**Response:**

Thank you for this insightful comment. We agree that the original wording overstated the evidential basis for the moderation claim. As you correctly note, the present study does not directly test whether typological distance moderates the relationship between language prestige and source language shining through. Rather, this interpretation was inferred from comparisons between our findings and those reported in previous studies involving different language pairs.

We considered the possibility of incorporating additional language pairs into the analysis; however, comparable bidirectional corpora were not available for a systematic cross-linguistic investigation. We therefore agree that the available evidence is insufficient to support a direct moderation claim.

To address this concern, we have carefully revised the relevant sections of the manuscript by substantially softening the original wording and reframing the argument as a theoretical interpretation rather than an empirically demonstrated effect. The revised text now presents typological distance as a plausible explanatory factor that may help account for variation in the strength of shining-through across language pairs, while explicitly acknowledging that a direct test of this proposition would require future research involving multiple language pairs and a more appropriate comparative design. Examples of the revision are provided below:

[Lines 35-40, Page 1]

A comparison with findings from studies involving typologically closer language pairs further suggests that typological distance may help explain variation in the influence of language prestige on source language shining through. Specifically, the prestige effect appears to be weaker when the source and target languages are typologically more distant, although this interpretation requires direct empirical testing in future research.

[Lines 909-929, Pages 45-46]

However, a comparison with the findings of Evert and Neumann [8] suggests a potentially interesting contrast: whereas the language prestige effect might be expected to produce an even stronger shining-through effect in English-to-Chinese translation than that observed for the typologically closer English-German pair, the present results do not indicate a comparatively stronger effect for Chinese. One possible, and admittedly speculative, interpretation is that the substantial typological distance between Chinese and English may attenuate the prestige effect to some extent, such that typological distance could act as a moderating factor in the relationship between language prestige and shining-through. More specifically, in the translation direction from a high-status language to a low-status one, the prestige effect is more likely to manifest when the source and target languages are typologically similar. However, when the source and target languages are typologically different, the prestige effect is likely to be relatively diminished. It should be emphasised, however, that this moderation is not directly tested in the present study. The present design does not include a typologically closer language pair for comparison within the same analytical framework, and the observation is based on a comparison across studies that differ in corpora, operationalisations and methods. As such, this interpretation should be regarded as a theoretically motivated hypothesis for future research, rather than as direct empirical evidence of a moderating effect.

[Lines 979-988, Page 48]

Firstly, while this study provides new evidence broadly consistent with the language prestige hypothesis and the risk-aversion hypothesis, it also raises the possibility, based on comparisons with previous studies involving typologically closer language pairs, that typological distance may influence the extent to which language prestige is reflected in source language shining through. Because this possibility was not directly tested in the present study, it should be regarded as a theoretical interpretation rather than an empirically established finding. Nevertheless, it points to a potentially fruitful direction for future research on the interaction between linguistic and sociolinguistic factors in translation.

Comment 2:

*A second concern involves the operationalization of language prestige. The study assumes English functions as a more prestigious language and Chinese as a less prestigious one within the proposed framework, but prestige is not formally defined or measured. Greater theoretical justification is needed. In particular, it would be useful to clarify whether prestige is treated as an assumed sociolinguistic property of the language pair or as an empirically measurable construct.*

**Response:**

Thank you for highlighting this important issue. We agree that the original manuscript did not provide sufficient justification for treating English as a language of higher global prestige and Chinese as a language of comparatively lower global prestige within the proposed framework.

To address this concern, we have substantially expanded the theoretical discussion of language prestige and clarified how the construct is operationalised in the present study. Specifically, language prestige is not treated as a variable that is directly measured in our dataset. Rather, it is conceptualised as a sociolinguistic property established in previous scholarship on global language hierarchies, language diffusion, and international communicative influence. In this sense, prestige is employed as a theoretically motivated explanatory construct rather than an empirically measured variable in the current analysis.

We consulted relevant studies on language prestige and the global status of English and Chinese. These studies suggest that, although Chinese has experienced a significant increase in international influence, English continues to occupy a dominant position in global communication, education, science, business, and international institutions (e.g., (Gil, 2021). Accordingly, the distinction adopted in this study is based on the relative global reach and institutional influence of the two languages rather than on the number of native speakers alone.

In the revised manuscript, we have incorporated a more detailed discussion of both language prestige and typological distance. We now explicitly define the notion of a global language with reference to established frameworks (Ammon, 2010; Crystal, 1997) and explain why English and Chinese occupy different positions within the contemporary global linguistic landscape. We also clarify that Chinese belongs to the Sino-Tibetan language family, whereas English is a Germanic language, thereby providing the theoretical basis for our discussion of typological distance. To improve transparency, this discussion has been added as a dedicated subsection that explains the conceptual and operational rationale underlying the study. The revised text reads as follows:

[Lines 278-333, Pages 13-15]

Taken together, previous research suggests that source language shining through is shaped by multiple interacting factors. Existing studies have highlighted the potential roles of language prestige, typological distance and register variation. However, several important questions remain unresolved. First, the relative influence of language prestige and typological distance has rarely been examined in language pairs that are both sociolinguistically asymmetric and typologically distant. Second, little is known about whether the interaction between these factors remains stable across different registers. Finally, most previous evidence has been derived from European language pairs, leaving the generalisability of existing explanations to English–Chinese translation largely unexplored. The present study addresses these gaps by examining shining-through effects in bidirectional English–Chinese translations across multiple registers within a unified analytical framework.

To enhance the interpretability and replicability of findings in such a context, it is necessary to clarify the typological and sociolinguistic profiles of the English–Chinese pair and to specify how the shining-through effect is operationally defined in this study. It should be emphasised that both typological distance and language prestige are best understood as continua rather than fixed categorical distinctions.

Typologically, English and Chinese belong to distinct language families [35]. English is a Germanic language within the Indo-European family, closely related to German and Dutch, and heavily influenced by French, a member of the Romance branch [36]. Despite their genealogical differences, the Germanic and Romance languages share a number of structural features, such as the existence of tense systems, definite and indefinite articles, and the grammatical distinction between singular and plural nouns [37]. These features are either absent or considerably less grammaticalised in Chinese, which belongs to the Sino-Tibetan (Sinitic) family [38]. Furthermore, unlike English, which employs an alphabetic writing system, Chinese is a logographic language that uses Chinese characters as written symbols. As a result, the structural distance between English and Chinese is far greater than that between typologically related European languages, encompassing profound differences in morphology, syntax and information structure, and involving minimal overlap in linguistic encoding strategies [35].

From a sociolinguistic perspective, both English and Chinese can be described as global languages, yet they occupy asymmetrical positions within the world linguistic system. In the present study, language prestige is treated as a theoretically motivated sociolinguistic property of the language pair, rather than as an empirically measured construct. This assumption is grounded in the established sociolinguistic literature on the asymmetric institutional positions occupied by English and Chinese in international communication [39-45]. Crystal [39, 422] defines a global language as one that holds a “special role that is recognised in every country”, while Ammon [40, 101-102] further distinguishes between a language’s global status (geographical coverage), global function (use in international communication) and the factors influencing that function (the sociopolitical and economic motivations for its global use). Although Chinese, in all its varieties, has the largest number of native speakers (over one billion), according to Eberhard [41], its native use is largely geographically concentrated in China [42]. English, by contrast, has a smaller native speaker base across the USA, Canada, Britain, Ireland, Australia, New Zealand, South Africa and parts of the Caribbean, but it enjoys broader global diffusion and institutional presence. As McArthur [43, 414] notes, “the Chinese language complex is much larger than that of native English users, but its members are largely ethnically and culturally homogeneous, and its worldwide distribution is limited”. Despite the growing international influence of Chinese, English continues to occupy a central position as the global lingua franca [44,45]. […]

References cited in the response:

Ammon, Ulrich. 2010. ‘World Languages: Trends and Futures’. In *The Handbook of Language and Globalization*, edited by Nikolas Coupland. John Wiley & Sons, Ltd. <https://doi.org/10.1002/9781444324068.ch4>.

Crystal, David. 1997. *English as a Global Language*. Cambridge University Press. <https://doi.org/10.1017/CBO9780511486999>.

Gil, Jeffrey. 2021. *The Rise of Chinese as a Global Language: Prospects and Obstacles*. Springer International Publishing. <https://doi.org/10.1007/978-3-030-76171-4>.

Comment 3:

*Relatedly, the criteria for selecting the 34 linguistic indicators could be explained more explicitly, particularly regarding their cross-linguistic comparability, theoretical motivation, and whether alternative feature sets were considered.*

**Response:**

Thank you for raising this important point. We agree that the original manuscript did not provide sufficient information regarding the rationale for selecting the 34 linguistic indicators. We have therefore substantially expanded the methodological description to clarify the criteria underlying feature selection.

Specifically, we now explain the theoretical motivation for including these indicators and their relevance to the investigation of source language shining through. We also clarify the extent to which the selected features are comparable across Chinese and English, and discuss the procedures adopted to ensure that the indicators capture analogous linguistic dimensions in the two languages despite their typological differences.

In addition, we have elaborated on the rationale for selecting these particular features over alternative linguistic measures. The revised manuscript now explains that the feature set was chosen to provide broad coverage of lexical, syntactic and discourse-level characteristics that have been widely employed in corpus-based and translation studies, while also ensuring sufficient cross-linguistic applicability and interpretability for comparative analyses.

To improve transparency and reproducibility, a dedicated explanation of the feature-selection process has been added to the Methods section. The revised text also clarifies how these indicators relate to previous studies and the theoretical framework adopted in the present research. An excerpt of the revision is provided below:

[Lines 478-515, Pages 23-25]

[…] The selection of linguistic indicators was guided by three criteria: theoretical relevance, cross-linguistic comparability and empirical interpretability. First, each feature was required to have a clear theoretical connection to one or more of the major typological distinctions between Chinese and English discussed above. Second, only features that could be operationalised in a broadly comparable manner across both languages were retained, ensuring that observed differences would reflect genuine linguistic variation rather than language-specific annotation artefacts. Third, the feature set was designed to capture lexical, syntactic and discourse-level dimensions of variation. On the basis of the typological distinctions outlined above, 34 linguistic variables were identified for the construction of the classification model, each of which is described in detail in Table 2.

With respect to parts of speech (POS), four categories of content words were incorporated into the analysis. Function words were excluded on the grounds that no consistent cross-linguistic correspondence exists between Chinese and English in this domain. Given that nouns and adjectives characteristically co-occur, as do verbs and adverbs [55] , these four content word categories collectively serve as the primary means of operationalising the dynamic–stative dichotomy between the two languages. The second and third sets of features relate to cohesive devices, specifically connectives and pronouns. Although both demonstrative and personal pronouns are attested in Chinese and English, the two languages do not share an identical taxonomy of conjunctions. A cross-linguistic comparison yielded five conjunction categories common to both languages which were employed to examine the hypotactic–paratactic dichotomy. The compact–diffusive dichotomy was investigated through two further feature sets: punctuation marks and syntactic structures. Within the domain of syntax, passive constructions were subject to dedicated analysis as the primary means of operationalising the passive–active dichotomy. Mean dependency distance, average sentence length and existential sentences were included to operate compact-diffusive dichotomies due to their comparability across both languages. Finally, classifiers and modal verbs were incorporated as variables to probe the synthetic–analytic distinction. Several additional features were considered but ultimately excluded. For example, tense markers were not included because they partially overlap with verbal morphology and would introduce redundancy into the feature space. Likewise, language-specific features (e.g., *ba*-construction in Chinese, articles and cases in English) were excluded because they lacked clear equivalents in the other language, thereby reducing the validity of cross-linguistic comparisons.

Comment 4:

*Methodologically, additional information is required regarding FDA implementation, model validation, hyperparameter selection, and cross-validation procedures to allow readers to fully evaluate the robustness and reproducibility of the analyses. Reporting effect sizes alongside significance tests would strengthen the interpretation of the results.*

**Response:**

Thank you for highlighting this issue. We agree that the original manuscript did not provide sufficient methodological detail regarding the implementation and validation of the Flexible Discriminant Analysis (FDA) model, nor did it adequately report effect sizes alongside the significance tests.

To address these concerns, we have substantially expanded the methodological description to improve the transparency, robustness and reproducibility of the analyses. Specifically, we now provide detailed information regarding the FDA implementation, including the model-fitting procedure, hyperparameter tuning strategy, cross-validation protocol and model validation process. The revised manuscript explains how the dataset was partitioned into training and test sets, how hyperparameters were optimised using cross-validation, and how the final model was evaluated on an independent held-out dataset.

In addition, we have clarified the rationale for the selected FDA specification and included further details regarding the interpretation of the discriminant dimensions. To facilitate reproducibility, the revised version also reports the optimal hyperparameter settings and the performance of the final model.

Regarding statistical reporting, we agree that significance tests alone do not fully convey the magnitude of observed effects. We have therefore added the corresponding effect size measures throughout the Results section and incorporated their interpretation where appropriate. This additional information allows readers to better evaluate the practical significance of the findings in addition to their statistical significance.

These revisions are intended to provide a more comprehensive account of the analytical procedures and to enable readers to more fully assess the robustness and reproducibility of the study. Examples of the revisions are provided below:

[Lines 554-577, Pages 28-29]

[…] In the present study, a minor methodological modification was introduced: LDA was replaced by FDA in order to accommodate a violation of the multivariate normality assumption in the dataset (Henze-Zirkler test: HZ = 1.06, *p* < 0.001). First proposed by Hastie et al. [57], FDA constitutes a non-parametric extension of LDA that employs a non-linear regression framework to achieve superior classification performance under conditions in which the assumptions of multivariate normality and homogeneity of covariance matrices are not satisfied. Prior to model training, the dataset was randomly partitioned into a training set (80%) and a held-out test set (20%). FDA was implemented in R using the *fda* method in the *caret* package, which employs Multivariate Adaptive Regression Splines (MARS) as the underlying regression engine. Hyperparameter tuning was conducted on the training set using 10-fold cross-validation. A grid search was performed over the MARS parameters degree and nprune, and the optimal parameter combination (degree = 1, nprune = 44) was selected based on cross-validated classification accuracy. The final FDA model was then fitted using these parameters and evaluated on the held-out test set. While MARS provided more flexible modelling of non-linear predictor-response relationships, it does not yield directly interpretable coefficients for individual predictors. Therefore, a supplementary FDA model was fitted using polynomial regression as the basis function, which produces a single linear coefficient for each predictor on each discriminant dimension. This specification allows for a more straightforward characterisation of the direction and relative magnitude of each variable’s contribution to the discriminant space and was used exclusively for the purpose of model interpretation, rather than classification.

[Lines 715-729, Pages 35-36]

A closer inspection of the density plot for dimension 1 reveals that both translated varieties were displaced from their respective originals toward a more central position. Wilcoxon Rank Sum tests confirmed that these displacements were statistically significant for both English-to-Chinese (*z* = 21.8, *p* < 0.001) and Chinese-to-English translations (*z* = 21.1, *p* < 0.001). To further examine the degree of distributional similarity between translated and original texts, kernel density overlap coefficients were computed: a lower overlap value indicates greater distributional divergence between a translated variety and its corresponding original, which may be interpreted as reflecting a stronger shining-through effect. English-to-Chinese translations showed an overlap of 0.26 with Chinese originals, compared to 0.31 for Chinese-to-English translations with English originals, suggesting that English-to-Chinese translations were distributionally more distant from their target-language baseline. This pattern may suggest a stronger shining-through effect in English-to-Chinese translations.

[Lines 836-861, Pages 41-42]

Fig 8a demonstrates a significant difference between Chinese and English in mean dependency distance (*ꭓ²* = 1098.59, *df* = 3, *p* < 0.001), with Chinese texts having higher values than English texts, and it also shows that original Chinese texts were characterised by a longer distance than translated Chinese texts. Fig 8b presents a comparison of adjectives, revealing that adjectives were used significantly more frequently in English (*ꭓ²* = 1500.25, *df* = 3, *p* < 0.001). The shining-through effect seems to be evident in this figure, as translated English from Chinese used adjectives less frequently than original English texts. Fig 8c further corroborates the hypotactic-paratactic dichotomy, showing that third-person pronouns were significantly less frequently employed in both translated and original Chinese texts (*ꭓ²* = 637.83, *df* = 3, *p* < 0.001). This indicator also demonstrates the shining-through effect, as evidenced by a higher frequency of third-person pronouns in translated Chinese and a lower frequency in translated English. Fig 8d illustrates that passive constructions were underrepresented in both translated and original Chinese texts (*ꭓ²* = 926.45, *df* = 3, *p* < 0.001). Similarly, the source language shining through effect is observable in this linguistic variable. Lastly, Fig 8e indicates a significantly higher frequency of collective classifiers/measure nouns in Chinese than in English texts (*ꭓ²* = 737.4, *df* = 3, *p* < 0.001). In summary, the analysis of these selected variables highlights the typological differences between Chinese and English, while also demonstrating how these differences contributed to the manifestation of source language shining through in both translation directions.

Comment 5:

*The interpretation of PCA components should also be presented cautiously, as PCA identifies variance structure rather than linguistic categories per se. Similarly, the interpretation of the first FDA dimension as representing typological distance and source-language influence should be treated cautiously. Discriminant dimensions and high classification accuracy are statistical measures of group separation; they demonstrate that the text categories are distinct, but they do not by themselves identify the specific linguistic mechanisms or cognitive behaviors driving that variation.*

**Response:**

Thank you for this valuable comment. We agree that the original manuscript did not sufficiently distinguish between statistical patterns identified by the analyses and the theoretical interpretations derived from them.

As you correctly note, PCA identifies underlying variance structures in the data rather than predefined linguistic categories, and FDA dimensions primarily reflect statistical separation among groups. Likewise, while the first FDA dimension was interpreted in relation to typological distance and source language shining through, the dimension itself does not constitute direct evidence of these underlying mechanisms. Similarly, high classification accuracy indicates that the text categories are distinguishable on the basis of the selected linguistic features, but it does not by itself reveal the specific linguistic processes or cognitive behaviours responsible for those differences.

To address this concern, we have carefully revised the relevant sections of the manuscript. We have softened the wording throughout and reframed the interpretations as theoretically informed explanations that are consistent with the observed statistical patterns, rather than as direct demonstrations of particular linguistic mechanisms or cognitive processes. In addition, we now explicitly acknowledge the exploratory nature of the PCA and FDA results and clarify the distinction between statistical discrimination and causal or cognitive interpretation.

These revisions are intended to provide a more cautious and methodologically rigorous interpretation of the findings while preserving the theoretical insights that emerge from the observed patterns. Examples of the revision are provided below:

[Lines 591-603, Pages 29-30]

[…] Along dimension 1 (Fig 1a), a clear separation between Chinese and English texts could be observed: both original and translated Chinese texts were positioned between 0 and +6, while original and translated English texts were located between -6 and 0. Examination of dimension 2 in Fig 1b seems to suggest that it was more closely associated with register variation. Specifically, journalistic and academic texts were predominantly situated in the negative coordinate range, whereas fictional texts were positioned in the opposite direction. General texts occupied a broader area, encompassing all three register categories in the biplot. On this basis, dimension 1 appears to correspond, at least in part, to the language-related distinction that is the focus of this study, although it should be noted that PCA dimensions reflect overall variance structure and may also be influenced by other, unmeasured sources of variation.

[Lines 720-729, Page 36]

[…] To further examine the degree of distributional similarity between translated and original texts, kernel density overlap coefficients were computed: a lower overlap value indicates greater distributional divergence between a translated variety and its corresponding original, which may be interpreted as reflecting a stronger shining-through effect. English-to-Chinese translations showed an overlap of 0.26 with Chinese originals, compared to 0.31 for Chinese-to-English translations with English originals, suggesting that English-to-Chinese translations were distributionally more distant from their target-language baseline. This pattern may suggest a stronger shining-through effect in English-to-Chinese translations.

Comment 6:

*The discussion occasionally overstates the findings, particularly when referring to universality and causal explanations. The authors should distinguish more clearly between empirical findings and theoretical interpretations.*

**Response:**

Thank you for this constructive suggestion. We agree that some parts of the original Discussion section did not sufficiently distinguish between the empirical findings of the study and the broader theoretical interpretations derived from them. As a result, several statements may have conveyed a stronger degree of certainty than was warranted by the evidence.

To address this concern, we carefully reviewed the Discussion section and revised a number of passages that could be interpreted as implying universality or causal relationships. In the revised manuscript, we have adopted more cautious wording and explicitly distinguished between findings that are directly supported by the data and interpretations that are intended to provide a theoretical explanation of the observed patterns.

More specifically, claims concerning translation universals, language prestige, typological distance and source language shining through have been reframed as theoretically informed interpretations that are consistent with the present findings rather than as universally applicable or causally established conclusions. We have also added clarifications regarding the scope and limitations of the study to avoid overgeneralisation beyond the language pair, corpus and analytical framework investigated.

These revisions are intended to ensure that the Discussion more accurately reflects the evidential status of the findings while preserving the broader theoretical implications of the study. An example of the revision is provided below:

[Lines 867-872, Page 43]

[…] Overall, the model constructed using 34 linguistic variables shared by Chinese and English achieved a high level of classification performance and provided a useful basis for exploring patterns associated with typological differences and source language shining through via dimensionality-reduction and visualisation techniques.

[…] In the density plot of the first FDA dimension, translated Chinese and English texts occupied positions intermediate between source-language and target-language varieties, a pattern that is consistent with the presence of source language shining through in both translation directions.

Comment 7:

*The manuscript is generally well written, though some sections, particularly the literature review, are dense and would benefit from clearer signposting.*

**Response:**

Thank you for this constructive suggestion. We agree that clearer signposting is particularly important in information-dense sections, such as the literature review, as it helps readers follow the development of the argument and understand how individual sections contribute to the overall research rationale.

To address this concern, we carefully revised the manuscript to improve its organisational structure and readability. In particular, we strengthened the transitions between sections, added clearer signposting statements to guide readers through the literature review, and refined the presentation of the research gap and the progression of the argument. We also introduced additional summary and linking sentences where appropriate to clarify how the discussion of previous studies leads to the research questions and objectives of the present study.

These revisions are intended to enhance the logical coherence and accessibility of the manuscript while preserving the depth and breadth of the literature review. Example of the revision are provided below:

[Lines 150-345, Pages 7-16]

The first theory closely related to the present study is the language prestige hypothesis.

[…]

Addressing this question requires consideration of another important explanation for the shining-through effect, namely the typological distance itself.

[…]

While the language prestige hypothesis attributes variation in shining-through to asymmetries in the sociolinguistic status of languages, another line of research emphasises the role of structural differences between languages. This perspective is commonly referred to as the typological distance hypothesis, which can be summarised as follows:

[…]

Moreover, even when language prestige and typological distance are taken into account, the manifestation of shining-through may vary considerably across communicative contexts and registers.

[…]

Taken together, previous research suggests that source language shining through is shaped by multiple interacting factors. Existing studies have highlighted the potential roles of language prestige, typological distance and register variation. However, several important questions remain unresolved. First, the relative influence of language prestige and typological distance has rarely been examined in language pairs that are both sociolinguistically asymmetric and typologically distant. Second, little is known about whether the interaction between these factors remains stable across different registers. Finally, most previous evidence has been derived from European language pairs, leaving the generalisability of existing explanations to English–Chinese translation largely unexplored. The present study addresses these gaps by examining shining-through effects in bidirectional English–Chinese translations across multiple registers within a unified analytical framework.

Comment 8:

*A more explicit discussion of limitations (e.g., translator variability, genre-specific effects, and the generalizability of findings beyond the PCCE corpus) would also improve transparency.*

**Response:**

Thank you for this constructive suggestion. We agree that a more explicit discussion of the study’s limitations would improve the transparency and interpretability of the findings.

To address this concern, we have expanded the limitations section in the revised manuscript and explicitly discussed several factors that may affect the scope and generalisability of the results. In particular, we now acknowledge the potential influence of translator variability, including differences in individual translation styles and decision-making processes that could not be examined within the current corpus-based design. We also discuss the possibility that the observed patterns may be partly shaped by genre-specific characteristics, despite our efforts to compare multiple registers.

Furthermore, we clarify that the findings are based on the PCCE corpus and therefore should be interpreted within the context of the corpus composition and language pair examined in the present study. We explicitly note that the extent to which the findings generalise to other corpora, translation settings, genres or language pairs remains an open question that warrants further investigation.

In addition to identifying these limitations, we outline several directions for future research that could help address them, including the use of additional corpora, broader cross-linguistic comparisons, and analyses that account for translator-level variation.

These revisions are intended to provide a more balanced interpretation of the findings and to clarify the boundaries within which the conclusions of the present study should be understood. An excerpt of the revision is provided below:

[Lines 961-988, Pages 47-48]

Several limitations of the present study should be acknowledged to guide future research. Firstly, while this study provides new evidence broadly consistent with the language prestige hypothesis and the risk-aversion hypothesis, it also raises the possibility, based on comparisons with previous studies involving typologically closer language pairs, that typological distance may influence the extent to which language prestige is reflected in source language shining through. Because this possibility was not directly tested in the present study, it should be regarded as a theoretical interpretation rather than an empirically established finding. Nevertheless, it points to a potentially fruitful direction for future research on the interaction between linguistic and sociolinguistic factors in translation. Secondly, although the corpus includes multiple registers, the observed patterns may still be partly influenced by genre-specific characteristics associated with the particular text types represented in the dataset. The extent to which these findings generalise to other genres therefore remains to be examined. Thirdly, the present corpus-based design does not allow for a systematic investigation of translator variability. Individual differences in translation experience, stylistic preferences and decision-making strategies may contribute to variation in source language shining through and warrant further study. Furthermore, additional research is needed to validate the findings in other Chinese–English comparable corpora beyond PCCE and across a wider range of typologically distant language pairs. Finally, incorporating a broader range of explanatory variables may provide a more comprehensive understanding of source language shining through. As noted by De Sutter and Lefer [20], linguistic choices in translation are shaped by multiple interacting factors, including temporality, spatiality, translator-related variables, and technological developments. Such a multifactorial perspective may offer valuable insights into both diachronic and synchronic variation in translated language.

Comment 9:

*Finally, the Data Availability Statement appears inconsistent with PLOS data-sharing requirements. The authors should revise this statement and clarify how readers will be able to access the underlying data supporting the analyses.*

**Response:**

Thank you for highlighting this issue. We agree that the original Data Availability Statement did not sufficiently clarify how readers would be able to access the data and materials underlying the analyses.

To address this concern, we have revised the Data Availability Statement to align with PLOS data-sharing requirements. Specifically, the dataset and R scripts used in the present study have been deposited in an Open Science Framework (OSF) repository to facilitate transparency and reproducibility. To preserve the integrity of the double-blind review process, identifying information associated with the repository has been temporarily anonymised during peer review.

The revised statement now clearly explains how the underlying data and analytical code will be made available to readers. Upon completion of the review process, the repository will be fully accessible through a permanent public link, allowing readers to reproduce the analyses and verify the reported findings.

These revisions are intended to ensure compliance with the journal’s data-sharing policy and to enhance the transparency and reproducibility of the study. An excerpt of the revised statement is provided below:

The datasets generated by the research during and/or analysed during the current study are available in the Dataverse repository, [https://doi.org/10.7910/DVN/205YXZ](https://osf.io/bau9w/overview?view_only=4d0810d7a1f54e6481d10e44e5711556)

Please note that the repository link provided is currently restricted to peer-review access and will be replaced with a publicly accessible version upon acceptance.

Comment 10:

*With substantial revision, clearer qualification of the theoretical claims, and improved methodological transparency, the manuscript has the potential to make a meaningful contribution to corpus-based translation studies.*

**Response:**

In summary, we would like to express our sincere gratitude for your thorough, thoughtful, and constructive review of our manuscript. Your insightful comments have helped us identify important areas for improvement and have significantly strengthened the quality, clarity, and methodological rigor of the study.

In response to your suggestions, we have undertaken substantial revisions throughout the manuscript. These revisions include a more cautious and clearly qualified presentation of the theoretical claims, expanded methodological descriptions to improve transparency and reproducibility, additional justification of key concepts and analytical procedures, and a more explicit discussion of the study’s limitations and scope of interpretation. We have also revised the data availability statement and enhanced the overall organisation and readability of the manuscript.

We believe that these changes have substantially improved the manuscript and have addressed the concerns raised during the review process. We greatly appreciate the time and expertise you have invested in evaluating our work. Should there be any remaining issues or aspects that require further clarification or revision, we would be grateful for the opportunity to address them and further improve the manuscript.

Thank you again for your valuable feedback and consideration.
