# Supplementary material for: Does language prestige hypothesis hold true across typologically distant languages? A corpus-based study comparing shining-through effect in English-Chinese bidirectional translations
Source: PLoS One. 2026 Jul 22;21(7):e0353687. doi: 10.1371/journal.pone.0353687 (PMC13390861; doi:10.1371/journal.pone.0353687)
Supplement: Peer review history

# Peer review: Decision Letter 0

**DOI:** 10.1371/journal.pone.0353687.r001

**Authors:** Valery Solovyev

10 Jun 2026

PONE-D-26-21657

Does language prestige hypothesis hold true across typologically distant languages? A corpus-based study comparing shining-through effect in English-Chinese bidirectional translations

PLOS One

Dear Dr. Pan,

Thank you for submitting your manuscript to PLOS ONE. After careful consideration, we feel that it has merit but does not fully meet PLOS ONE’s publication criteria as it currently stands. Therefore, we invite you to submit a revised version of the manuscript that addresses the points raised during the review process.

Please submit your revised manuscript by Jul 24 2026 11:59PM. If you will need more time than this to complete your revisions, please reply to this message or contact the journal office at plosone@plos.org. When you're ready to submit your revision, log on to https://www.editorialmanager.com/pone/ and select the 'Submissions Needing Revision' folder to locate your manuscript file.

Please include the following items when submitting your revised manuscript:

- A letter that responds to each point raised by the academic editor and reviewer(s). You should upload this letter as a separate file labeled 'Response to Reviewers'.
- A marked-up copy of your manuscript that highlights changes made to the original version. You should upload this as a separate file labeled 'Revised Manuscript with Track Changes'.
- An unmarked version of your revised paper without tracked changes. You should upload this as a separate file labeled 'Manuscript'.

If you would like to make changes to your financial disclosure, please include your updated statement in your cover letter. Guidelines for resubmitting your figure files are available below the reviewer comments at the end of this letter.

If applicable, we recommend that you deposit your laboratory protocols in protocols.io to enhance the reproducibility of your results. Protocols.io assigns your protocol its own identifier (DOI) so that it can be cited independently in the future. For instructions see: https://journals.plos.org/plosone/s/submission-guidelines#loc-laboratory-protocols. Additionally, PLOS ONE offers an option for publishing peer-reviewed Lab Protocol articles, which describe protocols hosted on protocols.io. Read more information on sharing protocols at https://plos.org/protocols?utm_medium=editorial-email&utm_source=authorletters&utm_campaign=protocols.

As the corresponding author, your ORCID iD is verified in the submission system and will appear in the published article. PLOS supports the use of ORCID, and we encourage all coauthors to register for an ORCID iD and use it as well. Please encourage your coauthors to verify their ORCID iD within the submission system before final acceptance, as unverified ORCID iDs will not appear in the published article. *Only*  the individual author can complete the verification step; PLOS staff *cannot*  verify ORCID iDs on behalf of authors.

We look forward to receiving your revised manuscript.

Kind regards,

Valery D. Solovyev, Ph.D.

Academic Editor

PLOS One

Journal requirements:

When submitting your revision, we need you to address these additional requirements.

1. Please ensure that your manuscript meets PLOS ONE's style requirements, including those for file naming. The PLOS ONE style templates can be found at

https://journals.plos.org/plosone/s/file?id=wjVg/PLOSOne_formatting_sample_main_body.pdf and

https://journals.plos.org/plosone/s/file?id=ba62/PLOSOne_formatting_sample_title_authors_affiliations.pdf

2. Please note that PLOS One has specific guidelines on code sharing for submissions in which author-generated code underpins the findings in the manuscript. In these cases, we expect all author-generated code to be made available without restrictions upon publication of the work. Please review our guidelines at https://journals.plos.org/plosone/s/materials-and-software-sharing#loc-sharing-code and ensure that your code is shared in a way that follows best practice and facilitates reproducibility and reuse.

3. Please ensure that you include a title page within your main document. You should list all authors and all affiliations as per our author instructions and clearly indicate the corresponding author.

4. In the online submission form you indicate that your data is not available for proprietary reasons and have provided a contact point for accessing this data. Please note that your current contact point is a co-author on this manuscript. According to our Data Policy, the contact point must not be an author on the manuscript and must be an institutional contact, ideally not an individual. Please revise your data statement to a non-author institutional point of contact, such as a data access or ethics committee, and send this to us via return email. Please also include contact information for the third party organization, and please include the full citation of where the data can be found.

5. We notice that your supplementary figures are uploaded with the file type 'Figure'. Please amend the file type to 'Supporting Information'. Please ensure that each Supporting Information file has a legend listed in the manuscript after the references list.

If the reviewer comments include a recommendation to cite specific previously published works, please review and evaluate these publications to determine whether they are relevant and should be cited. There is no requirement to cite these works unless the editor has indicated otherwise.

[Note: HTML markup is below. Please do not edit.]

Reviewers' comments:

Reviewer's Responses to Questions

**Comments to the Author**

1. Is the manuscript technically sound, and do the data support the conclusions?

The manuscript must describe a technically sound piece of scientific research with data that supports the conclusions. Experiments must have been conducted rigorously, with appropriate controls, replication, and sample sizes. The conclusions must be drawn appropriately based on the data presented.

Reviewer #1: Partly

**********

2. Has the statistical analysis been performed appropriately and rigorously?

Reviewer #1: Yes

**********

3. Have the authors made all data underlying the findings in their manuscript fully available?

The PLOS Data policy requires authors to make all data underlying the findings described in their manuscript fully available without restriction, with rare exception (please refer to the Data Availability Statement in the manuscript PDF file). The data should be provided as part of the manuscript or its supporting information, or deposited to a public repository. For example, in addition to summary statistics, the data points behind means, medians and variance measures should be available. If there are restrictions on publicly sharing data—e.g. participant privacy or use of data from a third party—those must be specified.

Reviewer #1: No

**********

4. Is the manuscript presented in an intelligible fashion and written in standard English?

PLOS ONE does not copyedit accepted manuscripts, so the language in submitted articles must be clear, correct, and unambiguous. Any typographical or grammatical errors should be corrected at revision, so please note any specific errors here.

Reviewer #1: Yes

**********

5. Review Comments to the Author

Please use the space provided to explain your answers to the questions above. You may also include additional comments for the author, including concerns about dual publication, research ethics, or publication ethics. (Please upload your review as an attachment if it exceeds 20,000 characters)

Reviewer #1: The manuscript addresses an important and theoretically interesting question concerning the interaction of language prestige, typological distance, and source-language shining-through in English-Chinese bidirectional translation. The use of a bidirectional parallel corpus and a multivariate analytical framework represents a valuable contribution to translation studies. The study presents substantial evidence that shining-through effects occur in both translation directions and that these effects vary across registers. The corpus design and the effort to identify cross-linguistically comparable features are commendable.

However, several important issues require clarification and revision before the manuscript can be considered for publication. The most significant concern is the claim that typological distance moderates the relationship between language prestige and shining-through. This moderation effect is not directly tested; instead, the argument is inferred through comparison with previous studies involving different language pairs. At present, the findings demonstrate directional differences in shining-through effects, but they do not permit a direct test of whether typological distance moderates the relationship between language prestige and source-language influence. The manuscript should either provide stronger evidence supporting this moderation claim or substantially soften and reframe it as a theoretical interpretation.

A second concern involves the operationalization of language prestige. The study assumes English functions as a more prestigious language and Chinese as a less prestigious one within the proposed framework, but prestige is not formally defined or measured. Greater theoretical justification is needed. In particular, it would be useful to clarify whether prestige is treated as an assumed sociolinguistic property of the language pair or as an empirically measurable construct. Relatedly, the criteria for selecting the 34 linguistic indicators could be explained more explicitly, particularly regarding their cross-linguistic comparability, theoretical motivation, and whether alternative feature sets were considered.

Methodologically, additional information is required regarding FDA implementation, model validation, hyperparameter selection, and cross-validation procedures to allow readers to fully evaluate the robustness and reproducibility of the analyses. Reporting effect sizes alongside significance tests would strengthen the interpretation of the results. The interpretation of PCA components should also be presented cautiously, as PCA identifies variance structure rather than linguistic categories per se. Similarly, the interpretation of the first FDA dimension as representing typological distance and source-language influence should be treated cautiously. Discriminant dimensions and high classification accuracy are statistical measures of group separation; they demonstrate that the text categories are distinct, but they do not by themselves identify the specific linguistic mechanisms or cognitive behaviors driving that variation.

The discussion occasionally overstates the findings, particularly when referring to universality and causal explanations. The authors should distinguish more clearly between empirical findings and theoretical interpretations. The manuscript is generally well written, though some sections, particularly the literature review, are dense and would benefit from clearer signposting. A more explicit discussion of limitations (e.g., translator variability, genre-specific effects, and the generalizability of findings beyond the PCCE corpus) would also improve transparency.

Finally, the Data Availability Statement appears inconsistent with PLOS data-sharing requirements. The authors should revise this statement and clarify how readers will be able to access the underlying data supporting the analyses.

With substantial revision, clearer qualification of the theoretical claims, and improved methodological transparency, the manuscript has the potential to make a meaningful contribution to corpus-based translation studies.

**********

6. PLOS authors have the option to publish the peer review history of their article (what does this mean?). If published, this will include your full peer review and any attached files.

If you choose “no”, your identity will remain anonymous but your review may still be made public.

**Do you want your identity to be public for this peer review?**  For information about this choice, including consent withdrawal, please see our Privacy Policy.

Reviewer #1: No

**********

[NOTE: If reviewer comments were submitted as an attachment file, they will be attached to this email and accessible via the submission site. Please log into your account, locate the manuscript record, and check for the action link "View Attachments". If this link does not appear, there are no attachment files.]

To ensure your figures meet our technical requirements, please review our figure guidelines: https://journals.plos.org/plosone/s/figures

You may also use PLOS’s free figure tool, NAAS, to help you prepare publication quality figures: https://journals.plos.org/plosone/s/figures#loc-tools-for-figure-preparation.

NAAS will assess whether your figures meet our technical requirements by comparing each figure against our figure specifications.

# Author comment: Author response to Decision Letter 1

**DOI:** 10.1371/journal.pone.0353687.r002

25 Jun 2026

Response Letter to the Editors’ and Reviewers’ Comments

Date: 23 June 2026

Manuscript Number: PONE-D-26-21657

Manuscript Title: Does language prestige hypothesis hold true across typologically distant languages? A corpus-based study comparing shining-through effect in English-Chinese bidirectional translations

Dear Editors and Reviewers,

We would like to express our sincere gratitude for the opportunity to submit a revised version of our manuscript titled “Does language prestige hypothesis hold true across typologically distant languages? A corpus-based study comparing shining-through effect in English-Chinese bidirectional translations” to PLOS ONE. We are deeply grateful for the time and effort invested by you and the reviewers in providing thoughtful feedback and constructive suggestions. These have been instrumental in helping us refine and strengthen our work.

In response to the reviewers’ suggestions and editorial guidance, we have made the following key revisions:

a. Strengthened the theoretical framing by clarifying the conceptualisation of key constructs, including language prestige and typological distance;

b. Enhanced methodological transparency and reproducibility by providing more detailed descriptions of feature selection, data preprocessing, FDA implementation, hyperparameter tuning, cross-validation procedures, model validation and statistical reporting;

c. Improved the interpretation of multivariate analyses by distinguishing more explicitly between statistical patterns and theoretical explanations;

d. Expanded the discussion of limitations and scope conditions.

We believe that these revisions have substantially improved the clarity, methodological rigor, theoretical precision and transparency of the manuscript. The revised version provides a more explicit account of the analytical procedures, a more carefully qualified interpretation of the findings, and a clearer distinction between empirical evidence and theoretical inference. As a result, the study offers a more robust and nuanced contribution to corpus-based translation studies and to ongoing discussions concerning source language shining through, language prestige and typological differences in translation. We hope that the revised manuscript now meets the expectations of the journal and we sincerely appreciate your consideration.

In accordance with the instructions outlined in your letter dated 6 June 2026, we have uploaded both a clean version of the revised manuscript and a marked-up version with all changes highlighted in dark blue using MS Word.

Please find enclosed our point-by-point response to the editors’ and reviewers’ comments. Each comment is numbered and reproduced in italicised red font. Our responses follow immediately in purple font, with corresponding excerpts from the revised manuscript presented in dark blue.

Thank you once again for your valuable feedback and continued consideration of our work.

Yours sincerely,

Author(s)

___________________________________________________________________________________

Comments from the Editorial Office

Comment 1

Please ensure that your manuscript meets PLOS ONE’s style requirements, including those for file naming.

Response:

Thanks for highlighting this issue. We have carefully reviewed the PLOS ONE style templates provided and revised the manuscript accordingly to ensure full compliance with the journal’s formatting requirements, including file naming conventions, heading styles and reference formatting.

Comment 2

Please note that PLOS One has specific guidelines on code sharing for submissions in which author-generated code underpins the findings in the manuscript. In these cases, we expect all author-generated code to be made available without restrictions upon publication of the work.

Response:

Thanks for pointing out this issue. We have reviewed the PLOS ONE guidelines on code sharing. All author-generated R code underlying the analyses reported in this manuscript has been made publicly available without restrictions. The code has been deposited at OSF: https://doi.org/10.7910/DVN/205YXZ and is fully documented to facilitate reproducibility and reuse. A reference to this repository has been included in the section of data availability revised manuscript. Please note that the repository link provided is currently restricted to peer-review access and will be replaced with a publicly accessible version upon acceptance.

Comment 3

Please ensure that you include a title page within your main document. You should list all authors and all affiliations as per our author instructions and clearly indicate the corresponding author.

Response:

Thanks for this comment. A title page has been included within the main document. It lists all authors, their respective affiliations and clearly indicates the corresponding author, in accordance with PLOS ONE author instructions.

Comment 4

In the online submission form you indicate that your data is not available for proprietary reasons and have provided a contact point for accessing this data. Please note that your current contact point is a co-author on this manuscript. According to our Data Policy, the contact point must not be an author on the manuscript and must be an institutional contact, ideally not an individual. Please revise your data statement to a non-author institutional point of contact, such as a data access or ethics committee, and send this to us via return email. Please also include contact information for the third party organization, and please include the full citation of where the data can be found.

Response:

We thank the editorial team for drawing our attention to this issue. We acknowledge that the original contact point listed was a co-author, which does not comply with PLOS ONE’s Data Policy. We have revised the Data Availability Statement accordingly. The data underlying this study are openly accessible in the OSF repository. Below is the revised Data availability statement:

The datasets generated by the research during and/or analysed during the current study are available in the OSF repository, https://doi.org/10.7910/DVN/205YXZ

Please note that the repository link provided is currently restricted to peer-review access and will be replaced with a publicly accessible version upon acceptance.

Comment 5

We notice that your supplementary figures are uploaded with the file type ‘Figure’. Please amend the file type to ‘Supporting Information’. Please ensure that each Supporting Information file has a legend listed in the manuscript after the references list.

Response:

Thank you for pointing out this issue. Upon reflection, we have decided to incorporate the supplementary figures directly into the main text as numbered figures, rather than retaining them as supporting information files. The figures have been repositioned and renumbered accordingly within the manuscript, and the corresponding in-text citations and captions have been updated to reflect this change.

___________________________________________________________________________________

Comments from Reviewer #1:

The manuscript addresses an important and theoretically interesting question concerning the interaction of language prestige, typological distance, and source-language shining-through in English-Chinese bidirectional translation. The use of a bidirectional parallel corpus and a multivariate analytical framework represents a valuable contribution to translation studies. The study presents substantial evidence that shining-through effects occur in both translation directions and that these effects vary across registers. The corpus design and the effort to identify cross-linguistically comparable features are commendable. However, several important issues require clarification and revision before the manuscript can be considered for publication.

Response:

We would like to express our sincere gratitude for the time and effort you dedicated to evaluating the initial version of our manuscript. We are deeply appreciative of your thorough and professional review, as well as your insightful suggestions for improvement, with which we fully agree. In light of your comments, we have substantially revised the manuscript to enhance its conceptual grounding, methodological transparency and analytical precision. The main revisions and additions are summarised as follows:

a. Strengthened the theoretical framing by clarifying the conceptualisation of key constructs, including language prestige and source language shining through, and by carefully qualifying claims concerning typological distance, translation universals and causal interpretations.

b. Enhanced methodological transparency and reproducibility by providing more detailed descriptions of feature selection, data preprocessing, FDA implementation, hyperparameter tuning, cross-validation procedures, model validation and statistical reporting.

c. Improved the interpretation of multivariate analyses by distinguishing more explicitly between statistical patterns and theoretical explanations.

d. Expanded the discussion of limitations and scope conditions, including translator variability, register-specific effects, corpus-related constraints and the generalisability of the findings beyond the PCCE corpus and the English–Chinese language pair.

Concerning your specific comments, we have numbered and quoted them in italicised red font. Our responses follow each comment in purple font, with modifications and additions from the manuscript presented in dark blue font below our responses.

Comment 1:

The most significant concern is the claim that typological distance moderates the relationship between language prestige and shining-through. This moderation effect is not directly tested; instead, the argument is inferred through comparison with previous studies involving different language pairs. At present, the findings demonstrate directional differences in shining-through effects, but they do not permit a direct test of whether typological distance moderates the relationship between language prestige and source-language influence. The manuscript should either provide stronger evidence supporting this moderation claim or substantially soften and reframe it as a theoretical interpretation.

Response:

Thank you for this insightful comment. We agree that the original wording overstated the evidential basis for the moderation claim. As you correctly note, the present study does not directly test whether typological distance moderates the relationship between language prestige and source language shining through. Rather, this interpretation was inferred from comparisons between our findings and those reported in previous studies involving different language pairs.

We considered the possibility of incorporating additional language pairs into the analysis; however, comparable bidirectional corpora were not available for a systematic cross-linguistic investigation. We therefore agree that the available evidence is insufficient to support a direct moderation claim.

To address this concern, we have carefully revised the relevant sections of the manuscript by substantially softening the original wording and reframing the argument as a theoretical interpretation rather than an empirically demonstrated effect. The revised text now presents typological distance as a plausible explanatory factor that may help account for variation in the strength of shining-through across language pairs, while explicitly acknowledging that a direct test of this proposition would require future research involving multiple language pairs and a more appropriate comparative design. Examples of the revision are provided below:

[Lines 35-40, Page 1]

A comparison with findings from studies involving typologically closer language pairs further suggests that typological distance may help explain variation in the influence of language prestige on source language shining through. Specifically, the prestige effect appears to be weaker when the source and target languages are typologically more distant, although this interpretation requires direct empirical testing in future research.

[Lines 909-929, Pages 45-46]

However, a comparison with the findings of Evert and Neumann [8] suggests a potentially interesting contrast: whereas the language prestige effect might be expected to produce an even stronger shining-through effect in English-to-Chinese translation than that observed for the typologically closer English-German pair, the present results do not indicate a comparatively stronger effect for Chinese. One possible, and admittedly speculative, interpretation is that the substantial typological distance between Chinese and English may attenuate the prestige effect to some extent, such that typological distance could act as a moderating factor in the relationship between language prestige and shining-through. More specifically, in the translation direction from a high-status language to a low-status one, the prestige effect is more likely to manifest when the source and target languages are typologically similar. However, when the source and target languages are typologically different, the prestige effect is likely to be relatively diminished. It should be emphasised, however, that this moderation is not directly tested in the present study. The present design does not include a typologically closer language pair for comparison within the same analytical framework, and the observation is based on a comparison across studies that differ in corpora, operationalisations and methods. As such, this interpretation should be regarded as a theoretically motivated hypothesis for future research, rather than as direct empirical evidence of a moderating effect.

[Lines 979-988, Page 48]

Firstly, while this study provides new evidence broadly consistent with the language prestige hypothesis and the risk-aversion hypothesis, it also raises the possibility, based on comparisons with previous studies involving typologically closer language pairs, that typological distance may influence the extent to which language prestige is reflected in source language shining through. Because this possibility was not directly tested in the present study, it should be regarded as a theoretical interpretation rather than an empirically established finding. Nevertheless, it points to a potentially fruitful direction for future research on the interaction between linguistic and sociolinguistic factors in translation.

Comment 2:

A second concern involves the operationalization of language prestige. The study assumes English functions as a more prestigious language and Chinese as a less prestigious one within the proposed framework, but prestige is not formally defined or measured. Greater theoretical justification is needed. In particular, it would be useful to clarify whether prestige is treated as an assumed sociolinguistic property of the language pair or as an empirically measurable construct.

Response:

Thank you for highlighting this important issue. We agree that the original manuscript did not provide sufficient justification for treating English as a language of higher global prestige and Chinese as a language of comparatively lower global prestige within the proposed framework.

To address this concern, we have substantially expanded the theoretical discussion of language prestige and clarified how the construct is operationalised in the present study. Specifically, language prestige is not treated as a variable that is directly measured in our dataset. Rather, it is conceptualised as a sociolinguistic property established in previous scholarship on global language hierarchies, language diffusion, and international communicative influence. In this sense, prestige is employed as a theoretically motivated explanatory construct rather than an empirically measured variable in the current analysis.

We consulted relevant studies on language prestige and the global status of English and Chinese. These studies suggest that, although Chinese has experienced a significant increase in international influence, English continues to occupy a dominant position in global communication, education, sci

Attachment Submitted filename: Response to Reviewers.docx

# Editor report: Decision Letter 1

**DOI:** 10.1371/journal.pone.0353687.r003

**Authors:** Valery Solovyev

29 Jun 2026

Does language prestige hypothesis hold true across typologically distant languages? A corpus-based study comparing shining-through effect in English-Chinese bidirectional translations

PONE-D-26-21657R1

Dear Dr. Pan,

We’re pleased to inform you that your manuscript has been judged scientifically suitable for publication and will be formally accepted for publication once it meets all outstanding technical requirements.

Within one week, you’ll receive an e-mail detailing the required amendments. When these have been addressed, you’ll receive a formal acceptance letter and your manuscript will be scheduled for publication.

An invoice will be generated when your article is formally accepted. Please note, if your institution has a publishing partnership with PLOS and your article meets the relevant criteria, all or part of your publication costs will be covered. Please make sure your user information is up-to-date by logging into Editorial Manager at Editorial Manager® and clicking the ‘Update My Information' link at the top of the page. For questions related to billing, please contact billing support.

If your institution or institutions have a press office, please notify them about your upcoming paper to help maximize its impact. If they’ll be preparing press materials, please inform our press team as soon as possible -- no later than 48 hours after receiving the formal acceptance. Your manuscript will remain under strict press embargo until 2 pm Eastern Time on the date of publication. For more information, please contact onepress@plos.org.

Kind regards,

Valery D. Solovyev, Ph.D.

Academic Editor

PLOS One

Additional Editor Comments (optional):

Reviewers' comments:

# Editor report: Acceptance letter

**DOI:** 10.1371/journal.pone.0353687.r004

**Authors:** Valery Solovyev

PONE-D-26-21657R1

PLOS One

Dear Dr. Pan,

I'm pleased to inform you that your manuscript has been deemed suitable for publication in PLOS One. Congratulations! Your manuscript is now being handed over to our production team.

At this stage, our production department will prepare your paper for publication. This includes ensuring the following:

* All references, tables, and figures are properly cited

* All relevant supporting information is included in the manuscript submission,

* There are no issues that prevent the paper from being properly typeset

You will receive further instructions from the production team, including instructions on how to review your proof when it is ready. Please keep in mind that we are working through a large volume of accepted articles, so please give us a few days to review your paper and let you know the next and final steps.

Lastly, if your institution or institutions have a press office, please let them know about your upcoming paper now to help maximize its impact. If they'll be preparing press materials, please inform our press team within the next 48 hours. Your manuscript will remain under strict press embargo until 2 pm Eastern Time on the date of publication. For more information, please contact onepress@plos.org.

You will receive an invoice from PLOS for your publication fee after your manuscript has reached the completed accept phase. If you receive an email requesting payment before acceptance or for any other service, this may be a phishing scheme. Learn how to identify phishing emails and protect your accounts at https://explore.plos.org/phishing.

If we can help with anything else, please email us at customercare@plos.org.

Thank you for submitting your work to PLOS ONE and supporting open access.

Kind regards,

PLOS ONE Editorial Office Staff

on behalf of

Professor Valery D. Solovyev

Academic Editor

PLOS One
